# *Meso*‐Crowned Porphyrin as a Dual Cavity Hybrid Macrocycle for Improving the Efficiency and Stability of Perovskite Solar Cells

**DOI:** 10.1002/advs.202522461

**Published:** 2026-01-15

**Authors:** Muhammad Ans, Murat Ebic, Rafał A. Grzelczak, Joanna Kruszyńska, Kostiantyn Nikiforow, Pankaj Yadav, Bartosz Szyszko, Seckin Akin, Daniel Prochowicz

**Affiliations:** ^1^ Institute of Physical Chemistry Polish Academy of Sciences Warsaw Poland; ^2^ Laboratory of Advanced Materials & Photovoltaics (LAMPs) Necmettin Erbakan University Konya Türkiye; ^3^ Faculty of Chemistry University of Wrocław Wrocław Poland; ^4^ Department of Solar Energy School of Energy Technology Pandit Deendayal Energy University Gandhinagar Gujarat India; ^5^ Department of Metallurgical and Materials Engineering Necmettin Erbakan University Konya Türkiye

**Keywords:** host–guest complexation, meso‐crowned porphyrin, passivation, perovskite solar cells, stability

## Abstract

Perovskite solar cells (PSCs) have achieved remarkable power conversion efficiencies (PCEs) and cost‐effective fabrication processes. However, defects in the bulk and interfaces of perovskite materials, as well as Li^+^ migration (especially in n–i–p regular architecture), which are used to enhance the conductivity and hole mobility of spiro‐OMeTAD, can significantly impact device performance and stability. Herein, we report a rationally designed *meso*‐crowned porphyrin derivative (**[12]‐C‐4POR**) featuring dual macrocyclic binding sites, i.e., a porphyrin core for undercoordinated Pb^2+^ and a crown ether unit selective for Li^+^ to suppress surface defects and mitigate lithium‐ion migration simultaneously. The incorporation of **[12]‐C‐4POR** into perovskite films significantly reduced the trap‐state density and suppressed non‐radiative recombination, leading to improved charge‐carrier dynamics. Devices treated with **[12]‐C‐4POR** delivered a champion PCE of 23.14%, surpassing the control device (21.6%), along with enhanced open‐circuit voltage (V_OC_) and fill factor (FF). More importantly, the passivated devices retained ∼95% of their initial PCE after 800 h of continuous operation, compared to ∼55% for the control. This study demonstrates a dual‐site host–guest passivation strategy as an effective route to improve both efficiency and operational stability of PSCs.

## Graphical abstract

This study demonstrates a dual‐functional porphyrin‐based passivation molecule, **[12]‐C‐4POR**, capable of simultaneously coordinating Pb^2+^ and Li^+^ ions through its porphyrin core and crown ether moiety, respectively. This molecule effectively reduces trap‐state density, suppresses non‐radiative recombination, and mitigates Li^+^ migration at the perovskite/HTL interface, which improves both efficiency and operational stability of PSCs.

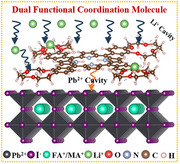

## 1 Introduction

Perovskite solar cells (PSCs) have received significant attention in the photovoltaic community due to their high efficiency, low‐cost fabrication processes, and the intrinsic advantages of perovskite materials [1, 2, 3]. Over the past decade, PSCs have depicted unprecedented development in power conversion efficiency (PCE) up to 27%, rivalling commercially available silicon‐based solar cells [4, 5, 6, 7]. However, the poor long‐term stability of PSCs still needs to be addressed for their commercial application [8, 9, 10]. The critical factors influencing the device stability are the formation of halide vacancies, undercoordinated Pb^2+^, and grain boundary dangling bonds, which are prevalent at the top interface of the perovskite film [11, 12, 13]. These defects, which are usually formed during thin film formation and post‐treatment processes, can cause charge recombination, ion migration, and fast degradation of PSCs [14]. To mitigate the aforementioned top‐interface perovskite defects, multiple strategies and solutions such as deposition of low‐dimensional perovskite overlayer [15, 16, 17, 18], treatment of the perovskite layer with ionic liquid [19, 20], and passivation of the perovskite film with a functional small molecule [21, 22, 23, 24, 25] have been developed. In the latter case, passivation agents are typically organic molecules containing Lewis base functional groups able to coordinate with undercoordinated Pb^2+^ ions, thereby passivating defects and suppressing nonradiative recombination in perovskite films [26, 27, 28, 29, 30]. Notably, molecular passivation enables precise control of defect state densities by selectively binding functional groups to unsaturated sites on the perovskite surface [31]. Besides, the instability of PSCs (especially in n–i–p regular architecture) can be induced by the migration of lithium ions (Li^+^), which are used to enhance the conductivity and hole mobility of spiro‐OMeTAD [32, 33]. Under operational conditions, Li^+^ is driven by the electric field from the adjacent hole transport layer (HTL) into the perovskite layer and electron transport layer (ETL), accelerating the device degradation and reducing efficiency [34, 35].

Recently, while developing a functional molecule for defect passivation, the researchers focused on macrocyclic compounds featuring coordination cavities tailored for binding various guest cations [36, 37, 38, 39, 40]. Among these, porphyrins, tetrapyrrolic macrocycles, are particularly noteworthy due to their rigid and preorganized coordination core, which is well‐suited for complexation with transition metal cations [41]. Porphyrins and metalloporphyrins have proven their ability to passivate perovskite surface defects and enhance charge carrier dynamics effectively [42]. For instance, Gao et al. used a series of D–*π*–A zinc(II)‐porphyrins functionalized with cyanoacrylic acid as an electron acceptor to passivate the perovskite surface and grain boundaries [43]. In turn, Zhang et al. reported on the D–*π*–A type zinc(II)‐porphyrin derivative equipped with the pyridine and triphenylamine units to increase the passivation effect of the undercoordinated Pb^2+^ ions in the perovskite film [44]. Metalloporphyrins with other functional groups have also shown great potential in improving the quality of perovskite thin films, thus enhancing the PCE and stability of the device [45, 46, 47, 48, 49]. On the other hand, metal‐free porphyrin molecules exhibit excellent coordination abilities to coordinate with Pb^2+^ in their inner core, which makes them an ideal agent for the passivation of the undercoordinated Pb^2+^ defects in the perovskite films [50, 51]. Therefore, careful design of metal‐free porphyrin with additional functionality may provide a promising strategy to further improve the performance and stability of PSCs.

**SCHEME 1:**
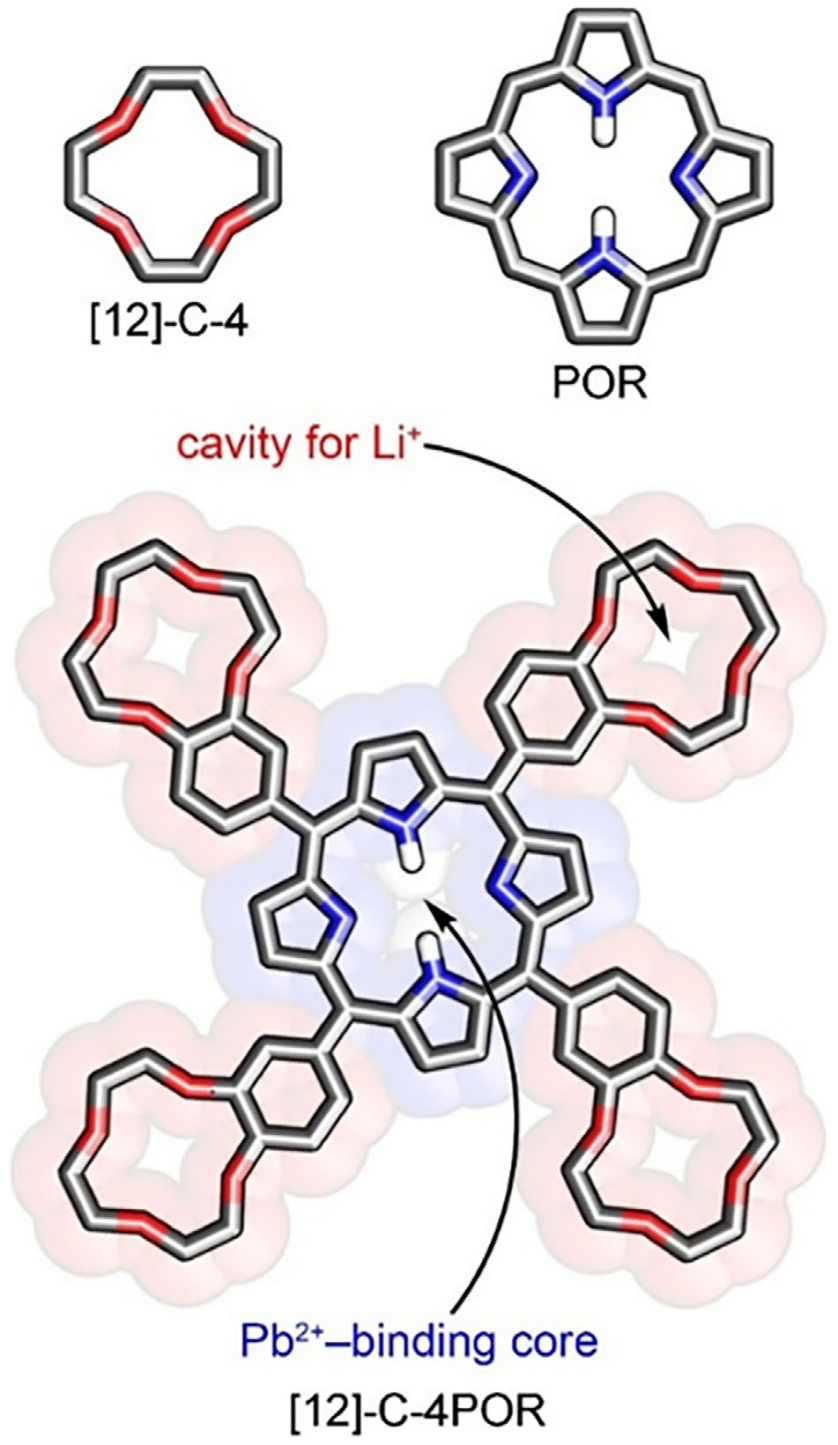
The *meso*‐crowned porphyrin—a dual cavity hybrid macrocycle preadapted for binding Pb^2+^ and Li^+^.

Herein, to address the challenge of simultaneous binding of Pb^2+^ and Li^+^ ions, we judiciously turned to *meso*‐crowned porphyrins—a class of porphyrins functionalized with crown ether moieties attached to the aromatic substituents at the methine bridges. These hybrid systems feature two distinct types of macrocyclic cavities: the porphyrin core and a crown ether unit, enabling site‐specific coordination of different metal cations [52, 53, 54, 55, 56, 57, 58, 59, 60, 61]. The [12]crown‐4 motifs were introduced at the peripheries of the porphyrin framework, to enable the effective capture of lithium ions (Scheme 1) [62]. This design enables a dual‐site coordination platform (further named as **[12]‐C‐4POR**) for binding Li^+^ within the crown ether cavity and Pb^2+^ in the porphyrin core. After treatment with an optimized amount of **[12]‐C‐4POR**, the perovskite films showed a suppressed nonradiative recombination and reduced surface trap density compared to the control film. These improvements lead to a maximum PCE of 23.14%, which outperforms that of the control device (21.6%). Moreover, the stability of devices with **[12]‐C‐4POR** treatment showed an improvement compared to the control cell, retaining ∼95% of the initial PCE after 800 h, whereas the control dropped to ∼55%. This study offers important insights into improving both the efficiency and durability of PSCs by employing a rationally designed passivation agent using a host–guest complexation strategy.

## 2 Results and Discussion

The macrocycle **[12]‐C‐4POR** was synthesized under conditions analogous to those reported by Krishnan for a related system [52]. The condensation of a crown‐ether‐functionalized benzaldehyde **S3** with pyrrole was conducted in propionic acid, following a modified Adler–Longo protocol (Scheme S1) [63]. Purification of the crude reaction mixture by flash column chromatography afforded macrocycle **[12]‐C‐4POR** in 15% yield (Figures S1–S3). The identity and purity of the product were confirmed by NMR spectroscopy (Figures S4–S11) and high‐resolution electrospray ionization mass spectrometry (HR‐ESI‐MS; Figure S12). The MS spectrum displayed a peak at *m/z* = 1199.4705 (calcd. 1199.4860), in agreement with the simulated isotopic pattern for [C_68_H_71_N_4_O_16_]^+^ (Figure S13). The ^1^H NMR spectrum of **[12]‐C‐4POR** exhibited features consistent with a highly symmetric aromatic macrocycle (Figure S4). Notably, the intracavity NH proton appeared as a sharp singlet at–2.82 ppm, characteristic of the strong shielding effect within the porphyrin cavity. In contrast, the β‐pyrrolic protons were deshielded, resonating at 8.87 ppm. The *meso*‐aryl substituents gave rise to three resonances in the 7.82–7.32 ppm region, while the methylene protons of the crown ether side chain appeared as a series of multiplets in the 4.51–3.91 ppm region.

Following its synthesis, macrocycle **[12]‐C‐4POR** was evaluated as a dual‐site ligand capable of coordinating both Pb^2+^ and Li^+^ ions in solution. To verify these complexation reactions, **[12]‐C‐4POR** was first reacted with lead(II) acetate trihydrate under reflux in methanol. After standard post‐synthetic work‐up, the lead(II) complex **[12]‐C‐4POR–Pb** was obtained (Figures S14–S17). Successful metallation was confirmed by ESI‐MS, which showed a signal corresponding to the lead(II)‐coordinated species (Figures S18–S19). The ^1^H NMR spectrum of the lead(II) porphyrin showed significant changes compared to the free‐base macrocycle. The NH resonance of the porphyrin cavity was absent in the spectrum recorded in [D]chloroform at 300 K, consistent with successful metal insertion within the porphyrin core. The β‐pyrrolic protons appeared as a singlet, shifted 0.11 ppm downfield relative to the free base, while the methylene protons of the crown ether showed resonances in the 5.52–3.85 ppm region. On the other hand, the addition of an excess of lithium bis(trifluoromethane)sulfonimide (Li‐TFSI) to a solution of **[12]‐C‐4POR** in [D_3_]acetonitrile resulted in a significantly altered ^1^H NMR spectral profile. Notably, the NH signal of the porphyrin cavity was observed across the entire range of lithium salt concentrations examined (Figure S20). Upon the addition of solid Li‐TFSI, the β‐pyrrolic proton resonance underwent a minimal 0.03 ppm downfield shift to 8.95 ppm, accompanied by minor changes in the aromatic region. The most pronounced effect of Li^+^ binding was observed in the crown ether segment of the macrocycle. The methylene proton resonances, initially located at 4.49, 4.34, 4.06, and 3.94 ppm, shifted to 4.65, 4.49, 4.03, and 3.91 ppm, consistent with the coordination of Li^+^ within the crown ether cavities. In contrast, the NH signal was only slightly affected, experiencing an upfield shift to −2.91 ppm, suggesting that lithium coordination occurs at the crown ether site, with no influence on the porphyrin N_4_ core. Finally, when lithium salt was added to a DCM/MeOH solution of **[12]‐C‐4POR–Pb**, the ESI‐MS spectrum showed a peak at *m/z* = 1411.4368 (calcd. 1411.4562), corresponding to a heterobimetallic species containing both Pb^2+^ and Li^+^ ions. Comparison of the experimental and simulated isotopic patterns confirmed the formation of a dual Pb^2+^/Li^+^ complex (Figure S21). Thus, it seemed justified to choose **[12]‐C‐4POR** as the passivation agent on the top surface of perovskite films to achieve complexation with the undercoordinated Pb^2+^ ions and inhibit Li^+^ diffusion.

**FIGURE 1:**
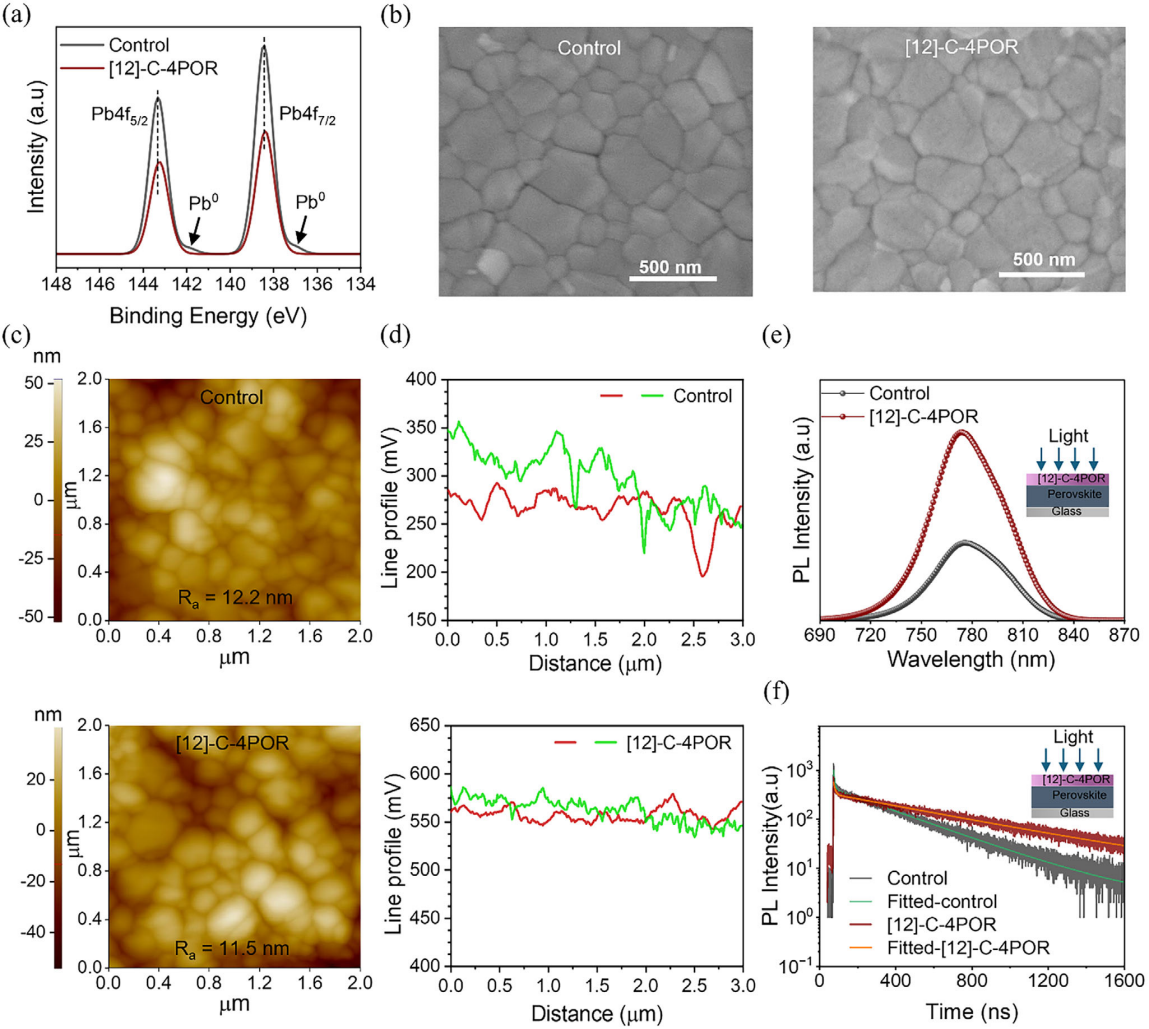
(a) Pb XPS spectra of the control and [12]‐C‐4POR‐based perovskite films. (b) SEM and (c) AFM images of the control and [12]‐C‐4POR‐based perovskite films. (d) Surface potential curve (KPFM) of the control and [12]‐C‐4POR‐based perovskite films (Green and red curves represent potential along the diagonal and horizontal of the surface, respectively, as shown in Figure S26). (e) PL and (f) TRPL spectra of the control and [12]‐C‐4POR‐based perovskite films.

To further validate the interaction between **[12]‐C‐4POR** and perovskite, we performed X‐ray photoelectron spectroscopy (XPS) on films to probe the presence of **[** **12]‐C‐4POR** on the surface of perovskite and to experimentally characterize their chemical interaction with the undercoordinated Pb^2+^ (Figure S22). The presence of higher C 1s content in the passivated perovskite film is indicative of the existence of **[12]‐C‐4POR** on the surface of perovskite (see Tables S1‐S3). In turn, the shift of the characteristic peak of Pb 4f (Figure 1a) and I 3d (Figure S23a) toward lower binding energies indicates an increase in the electron‐cloud density surrounding the Pb and I atoms. In addition, the metallic Pb^0^ vanishes after the treatment with **[12]‐C‐4POR**. On the other hand, the binding energy O 1s shifted toward higher values, indicating a decrease in electron cloud density around oxygen atoms (Figure S23b). Unfortunately, it was hard to make any conclusion about the possible interaction of Pb with porphyrin nitrogen atoms due to the similar binding energy of nitrogen bonds from perovskite and **[12]‐C‐4POR**, causing overlaps of the peaks on N1s spectrum (Figure S23c). These results suggest that **[12]‐C‐4POR** can react with the perovskite film likely through the host–guest interaction between the ethereal oxygen atoms and/or porphyrin nitrogen on **[12]‐C‐4POR** and Pb^2+^ ions on the perovskite surface [51, 64]. However, it is reasonable to expect multiple coordination modes for complexes arising from the interaction between the dual‐cavity macrocycle and Li^+^ and Pb^2+^ cations. To confirm the possibility of forming coordination compounds in which a single **[12]‐C‐4POR** molecule interacts with more than one lithium cation, we performed ESI‐MS measurements. The spectra were recorded for a DMSO/MeCN/DCM solution of **[12]‐C‐4‐POR** with the addition of LiOTf. As shown in Figure S24, multiple signals were detected in the ESI‐MS spectrum, including those corresponding to complexes containing four, three, two, or one lithium cation. We believe that determining the exact coordination mode in such a system is very challenging, as numerous types of complexes may form, including oligo‐ or polymeric species in which the crown‐ether units of two separate molecules sandwich one or more lithium or lead(II) cations.

To further explore the impact of **[12]‐C‐4POR** on the structural characteristics of perovskite films, scanning electron microscopy (SEM) was studied. Figure 1b shows that both the control and **[12]‐C‐4POR** passivated films exhibit similar grain morphology with negligible change in film uniformity and crystallinity upon passivation. The surface morphology and nanoscale roughness of the perovskite films were further investigated by atomic force microscopy (AFM), as shown in Figure 1c and Figure S25. Both the control and **[12]‐C‐4POR**‐treated films exhibited compact and pinhole‐free surfaces with densely packed grains, consistent with the SEM results. The average roughness (R_a_) of the control film was determined to be 12.2 nm, while that of the **[12]‐C‐4POR**‐modified film slightly decreased to 11.5 nm, indicating that the incorporation of **[12]‐C‐4POR** does not perturb the surface morphology. To gain further insight into the surface potential distribution and interfacial electronic environment, Kelvin probe force microscopy (KPFM) measurements were conducted (Figure 1d; Figure S26). The control film exhibited a surface potential centred around 250–300 mV with notable fluctuations across the scanned area, suggesting uneven charge distribution and localized trap states. In contrast, the **[12]‐C‐4POR**‐modified film displayed a significantly higher and more uniform surface potential (∼550 mV), implying reduced electronic disorder and enhanced Fermi‐level alignment at the surface. The narrower potential distribution and smoother line profile confirm that **[12]‐C‐4POR** effectively passivates surface defects and mitigates interfacial potential fluctuations. The improvement in potential uniformity is expected to facilitate balanced charge extraction and suppress nonradiative recombination losses in the resulting perovskite devices [65].

To further understand the effect of passivation on the charge carrier recombination dynamics, the steady‐state photoluminescence (PL) and time‐resolved photoluminescence (TRPL) spectra were collected. The **[12]‐C‐4POR** treated film shows a markedly higher PL intensity compared to the control sample, indicating a significant reduction in nonradiative recombination due to improved defect passivation (Figure 1e). The TRPL of the studied perovskite films was further performed, as shown in Figure 1f. The **[12]‐C‐4POR**‐based perovskite film exhibits a longer average carrier lifetime τ_ave_ of 585.30 ns than the control film (304.48 ns). The obtained TRPL decay spectra were fitted using the bi‐exponential function, and the fitting parameters are summarized in Table S4. The improved carrier lifetime in the **[12]‐C‐4POR‐**based perovskite film indicates that the trap‐assisted recombination at the perovskite surface was effectively suppressed [66, 67].

**FIGURE 2:**
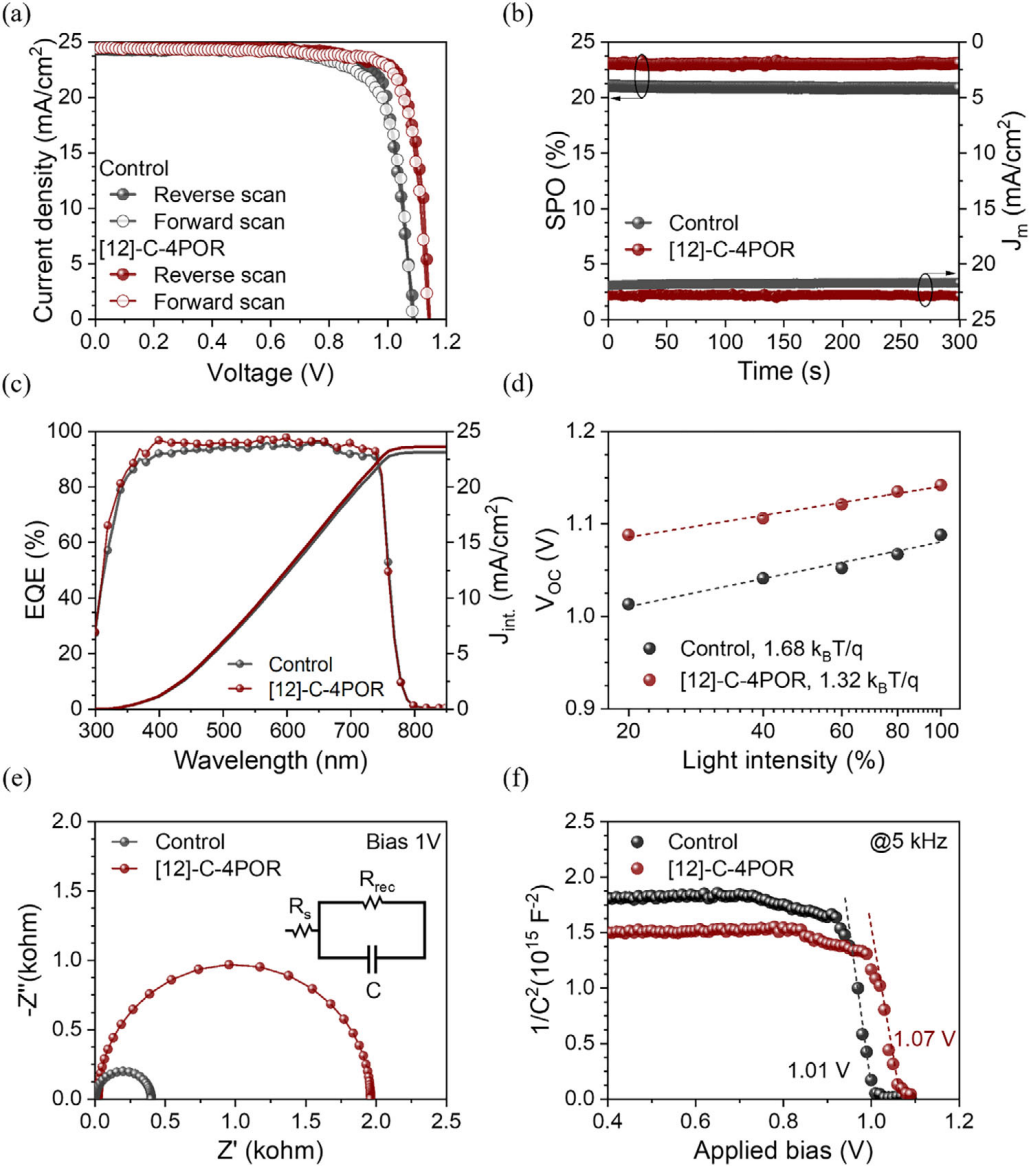
(a) *J–V* curves of the best performing control and [12]‐C‐4POR‐based PSCs under reverse and forward scans. (b) Steady‐state PCE of the control and [12]‐C‐4POR‐based devices. (c) EQE and the integrated J_SC_ curves of the control and [12]‐C‐4POR‐based PSCs. (d) Light intensity dependence of V_OC_ under reverse scanning. (e) Nyquist plots of impedance spectra for the control and [12]‐C‐4POR‐incorporated devices under 1 V bias. (f) Mott–Schottky plots recorded at 5 kHz for the control and [12]‐C‐4POR‐based devices.

To evaluate the photovoltaic performance and stability of PSCs incorporating **[12]‐C‐4POR**, we fabricated regular planar devices with an architecture of FTO/SnO_2_/Cs_0.05_(FA_0.90_MA_0.10_)_0.95_Pb(I_0.90_Br_0.10_)_3_/[12]‐C‐4POR/Spiro‐OMeTAD)/Au. The concentration of the **[12]‐C‐4POR** treatment solution was optimized with respect to photovoltaic performance (see details in Figure S27 and Table S5) and was determined to be 0.5 mg mL^−1^. The statistical analysis of photovoltaic parameters for 10 devices based on different concentrations is depicted in Figure S28. It can be seen that the optimized **[12]‐C‐4POR**‐based devices reveal enhanced photovoltaic parameters such as PCE, *J*_*SC*_, V_OC,_ and FF. The current density−voltage (*J−V*) curves of champion PSCs with and without **[12]‐C‐4POR** treatment are shown in Figure 2a. As seen, the control device exhibited a PCE of 21.26% under AM 1.5G irradiation at 100 mW cm^−2^ with an V_OC_ of 1.09 V, a *J*_*SC*_ of 24.31 mA cm^−2^, and a fill factor (FF) of 79.85%. In comparison, the best‐performing **[12]‐C‐4POR** device shows a maximum PCE of 23.14% with a *J*_*SC*_ of 24.72 mA cm^−2^, a V_OC_ of 1.150 V, and a FF of 81.59 %. At a steady‐state output measured at the maximum power point (MPP) over 300 s, the control and **[12]‐C‐4POR** device maintained a stable PCE of 21.26% (with minimal efficiency decay of 0.05%) and 23.14% (with minimal efficiency decay of 0.04 %), respectively (Figure 2b). The photovoltaic metrics shown in Table S6 reveal that PSCs with **[12]‐C‐4POR** demonstrated an overall performance improvement compared with control devices (for detailed comparison between our results and those previously reported for PSCs passivated by only crown ethers or porphyrins, see Table S7). The increment is mainly due to an increase in V_OC_ from 1.09 V to 1.15 V, which could be due to the suppressed non‐radiative recombination at the perovskite/HTM interface. Figure 2c illustrates the external quantum efficiency (EQE) spectra and the integrated current density of the control and **[12]‐C‐4POR**‐based devices. The calculated integrated *J*_*SC*_ are found to be 23.13 mA cm^−2^ and 23.62 mA cm^−2^, respectively, which are consistent with the values extracted from the *J–V* measurements.

To identify the charge recombination in PSCs, the ideality factor (*n*) fitted by the light intensity dependence of the V_OC_ curve is usually used. The value of *n* was calculated with the following equation:

(1) $$V_{oc}\left(P\right)=\frac{nkT}{q}\ln\left(P\right)+C$$

where k is the Boltzmann constant, T is the absolute temperature, and q is the elementary charge. As shown in Figure 2d, the **[12]‐C‐4POR**‐based device exhibits the smallest k_B_T/q slope (*n* = 1.32) than the control device (*n* = 1.68). This indicates the reduction of charge recombination processes and signifies the increase in V_OC_ in **[12]‐C‐4POR**‐based devices [68]. Furthermore, electrochemical impedance spectroscopy (EIS) was used to study the charge transfer and recombination of the devices (Figure 2e). The Nyquist plots were obtained at an applied voltage of 0.6 V, 0.8 V, and 1 V in darkness and fitted with the equivalent circuit diagram shown in Figure 2e and Figure S29. Table S8 provides the calculated values of recombination resistance (R_rec_) and series resistance (R_s_) for a given device. As seen, the device with a passivated perovskite film shows a higher R_rec_ compared to the control device, indicating an efficient suppression of charge recombination. Mott–Schottky (MS) analysis was performed to elucidate the enhancement of VOC and built‐in potential (V_bi_) of the control and passivated devices. The control device exhibits a V_bi_ of approximately 1.01 V, while the **[12]‐C‐4POR**‐modified device shows a slightly higher V_bi_ of 1.07 V. This increase in built‐in potential reflects an improved interfacial electric field (leading to a high FF) and increased V_OC_, which could facilitate more efficient charge separation and collection. To further investigate the effect of **[12]‐C‐4POR** on trap density (n_trap_), space charge limited current (SCLC) measurements on a hole‐only device with the structure FTO/NiOx/perovskite (with or without **[12]‐C‐4POR** treatment)/Spiro‐OMeTAD/Au under dark conditions, were carried out (Figure S30). Figure S30 displays the typical dark *J–V* characteristics alongside the calculated trap‐filled limit voltage (V_TFL_) values for all examined devices. The V_TFL_ of the passivated film with **[12]‐C‐4POR** is 0.35 V, which is lower than the control device (0.43 V). By using V_TFL_ values, the trap density was calculated using the equation of

(2) $$n_{t}=\frac{2\varepsilon \varepsilon_{o}V_{TFL}}{L^{2}e}$$

where ε_o_ is the vacuum permittivity, ε is the relative dielectric constant, e is the elementary charge, n_t_ is the trap‐state density, and L is the thickness of the perovskite films [69]. The n_t_ of the control device was 8.55 × 10^15^ cm^−3^, while the passivated device showed a lower value of 6.96 × 10^15^ cm^−3^. This reduction indicates effective defect passivation, which suppresses non‐radiative recombination and leads to improvements in both the V_OC_ and FF.

**FIGURE 3:**
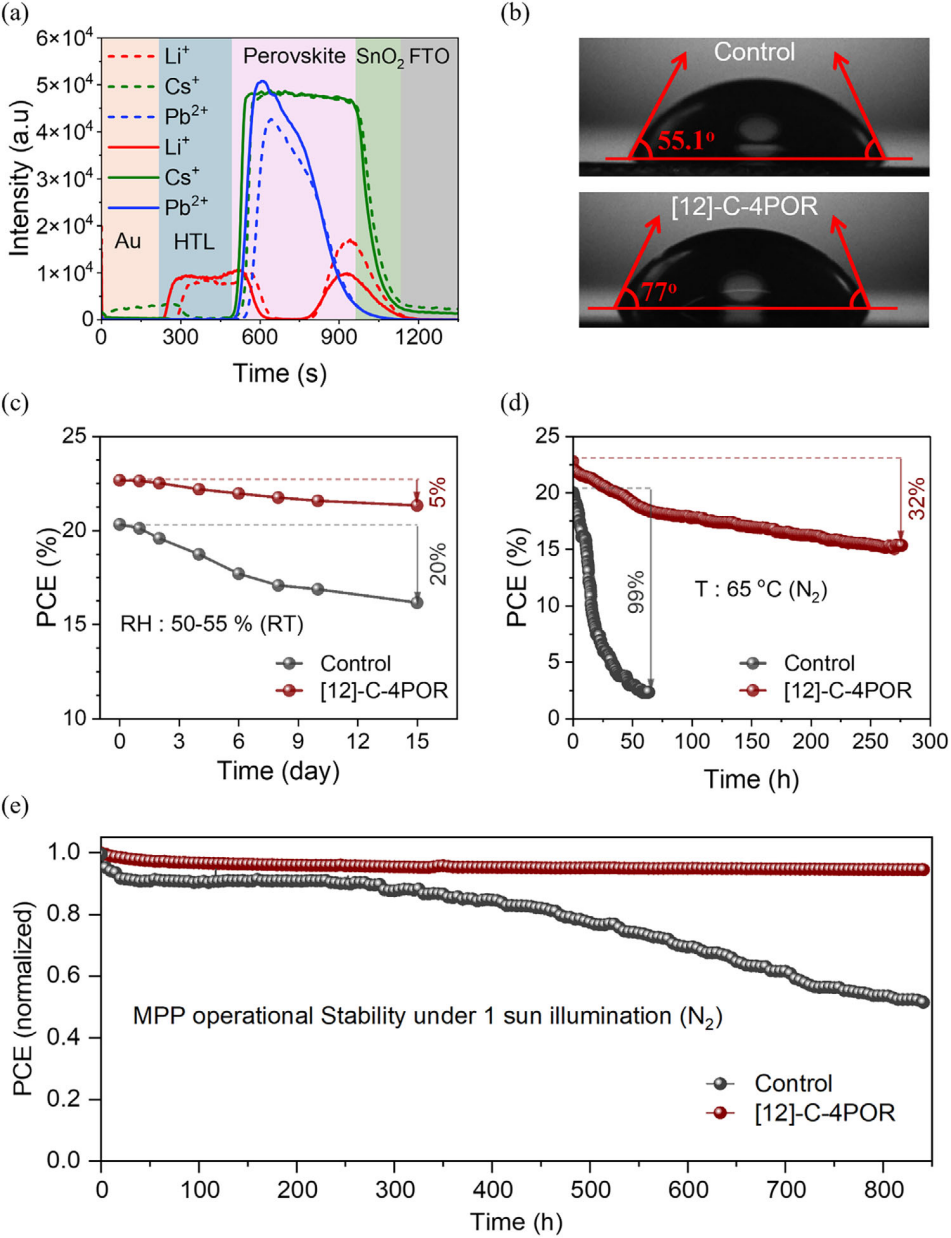
(a) TOF‐SIMS of the control (dashed line) and aged [12]‐C‐4POR (solid lines) based devices. (b) Static contact angle measurements of a water drop on top of the perovskite films. (c) Shelf‐life stability of the control and [12]‐C‐4POR‐based devices under daylight with RH ∼55%. (d) Thermal stability of the control and [12]‐C‐4POR‐based devices at 65°C under N_2_ atmosphere. (e) Operational stability of the control and [12]‐C‐4POR‐based devices under MPP tracking with continuous 1 sun illumination under a N_2_ atmosphere at room temperature.

Long‐term stability remains one of the major hurdles for advancing PSCs toward practical applications. Degradation under heat, light, and environmental stress can severely limit device performance and lifetime. First, we employed time‐of‐flight secondary ion mass spectrometry (TOF‐SIMS) to probe the intrinsic stability of the PSCs by examining the cross‐sectional distribution of Li^+^, Cs^+^, and Pb^2+^ as shown in Figure 3a. In the control device, a considerable amount of Li^+^ migrated from HTL to the perovskite layer/electron transport layer interface, which can create charged defects and disturb lattice stability. In contrast, the aged **[12]‐C‐4POR**‐based device showed much lower levels of Li^+^ migration, with only limited diffusion compared to the control device. This improvement could be attributed to the trapping of Li^+^ in the ether cavity, which effectively suppresses their movement. Additionally, the presence of **[12]‐C‐4POR** can enhance the moisture resistance of the perovskite/HTL interface due to the increase in the hydrophobic nature of **[12]‐C‐4POR**. The hydrophobicity of the control and **[12]‐C‐4POR**‐treated films was measured by the static contact angle of water droplets (Figure 3b). The passivated films depicted a higher contact angle (77 degrees) than that of the control (55 degrees), indicating enhanced water resistance. As a result, **[12]‐C‐4POR**‐based device exhibits improved shelf‐life stability under daylight with RH ∼ 55%. As shown in Figure 3c, **[12]‐C‐4POR**‐based device retained more than 94% of its initial efficiency, demonstrating its superior environmental stability compared to the control device (for the *J–V* characteristics of the respective devices measured after 15 days of shelf‐life stability see Figure S31). Next, we tested the thermal stability of the devices by keeping them at 65°C for 275 h in an inert nitrogen (N_2_) atmosphere (Figure 3d). As seen, the **[12]‐C‐4POR**‐treated device retained 70% of its initial performance after 275 h, while the control device completely degraded under the same conditions. Finally, the operational stability of PSCs was evaluated under continuous MPP tracking, as shown in Figure 3e. The control device exhibited a rapid degradation, with the normalized PCE dropping to ∼55% of its initial value after 800 h. In contrast, the device incorporating **[12]‐C‐4POR** maintained ∼95% of its initial PCE, demonstrating a remarkable improvement in long‐term stability. The improvement in the device stability highlights the key role of surface defect passivation by binding with undercoordinated Pb^2+^ and the capture of Li^+^ by the ether cavity, which together suppresses electric field induced degradation and reduces efficiency.

## 3 Conclusions

In conclusion, we have developed a dual‐functional porphyrin‐based passivation molecule, **[12]‐C‐4POR**, capable of simultaneously coordinating Pb^2+^ and Li^+^ ions through its porphyrin core and crown ether moiety, respectively. We found that porphyrin is strongly bonded with Pb^2+^, while the ether part captures the Li^+^ and suppresses its migration. The passivation with **[12]‐C‐4POR** effectively reduces trap‐state density, suppresses non‐radiative recombination, and mitigates Li^+^ migration at the perovskite/HTL interface. Furthermore, high hydrophobicity prevents the moisture content from reaching the perovskite. As a result, **[12]‐C‐4POR**‐treated PSCs achieved a PCE of 23.14% and exhibited outstanding operational stability, maintaining ∼95% of their initial efficiency after 800 h, while control devices suffered significant degradation. Furthermore, **[12]‐C‐4POR**‐treated device showed significantly better thermal and environmental stabilities compared to the control cell. These findings highlight the potential of molecularly engineered macrocyclic systems as versatile passivation agents to address both efficiency and stability challenges in next‐generation PSCs.

## Conflicts of Interest

The authors declare no conflicts of interest.

## Supporting information




**Supporting File**: advs73859‐sup‐0001‐SuppMat.docx.

## Data Availability

The data that support the findings of this study are available from the corresponding author upon reasonable request.
