# Supplementary material for: Meso‐Crowned Porphyrin as a Dual Cavity Hybrid Macrocycle for Improving the Efficiency and Stability of Perovskite Solar Cells
Source: Adv Sci (Weinh). 2026 Jan 15;13(10):e22461. doi: 10.1002/advs.202522461 (PMC12915086; doi:10.1002/advs.202522461)
Supplement: Supplementary file 1 — Supporting File: advs73859‐sup‐0001‐SuppMat.docx. [file ADVS-13-e22461-s001.docx]

**Supporting Information**

***Meso*-Crowned Porphyrin as a Dual Cavity Hybrid Macrocycle for Improving the Efficiency and Stability of Perovskite Solar Cells**

Muhammad Ans, Murat Ebic, Rafał A. Grzelczak, Joanna Kruszyńska, Kostiantyn Nikiforow, Pankaj Yadav, Bartosz Szyszko, Seckin Akin,^*^ and Daniel Prochowicz^*^

M. Ans, J. Kruszyńska, K. Nikiforow, D. Prochowicz

Institute of Physical Chemistry, Polish Academy of Sciences, Kasprzaka 44/52, Warsaw 01-224, Poland

E-mail: [dprochowicz@ichf.edu.pl](mailto:dprochowicz@ichf.edu.pl)

M. Ebic, S. Akin

Laboratory of Advanced Materials & Photovoltaics (LAMPs), Necmettin Erbakan University, Konya, 42090, Türkiye

E-mail: seckinakin@erbakan.edu.tr

R. A. Grzelczak, B. Szyszko

Faculty of Chemistry, University of Wrocław, 14 F. Joliot-Curie St., 50-383 Wrocław, Poland

P. Yadav

Department of Solar Energy, School of Energy Technology, Pandit Deendayal Energy University, Gandhinagar, 382007, Gujarat, India

S. Akin

Department of Metallurgical and Materials Engineering, Necmettin Erbakan University, Konya, 42090, Türkiye

**Table of contents**

| **Title** | **Page** |
| --- | --- |
| **Section 1. Experimental procedures** | **S5** |
| Material | **S5** |
| Device fabrication | **S5** |
| Current Density−Voltage Measurements | **S5** |
| X-Ray Photoelectron Spectroscopy (XPS) | **S5** |
| Scanning Electron Microscopy (SEM) | **S6** |
| Contact angle measurements | **S6** |
| Time-Resolved Photoluminescence (TRPL) | **S6** |
| External Quantum Efficiency (EQE) | **S6** |
| Electrochemical Impedance Spectroscopy (EIS) | **S6** |
| Atomic Force Microscopy (AFM) | **S6** |
| **Section 2. Results and discussion** | **S6** |
| NMR spectroscopy | **S6** |
| Mass spectrometry | **S6** |
| **Scheme 1**. The synthesis of porphyrin [12]-C-4POR | **S7** |
| Synthesis of S3@ under modified literature conditions.[1] | **S7** |
| **Figure S1**. The ^1^H NMR spectrum of **S3** (500 MHz, [D]chloroform, 300 K). | **S8** |
| **Figure S2**. The aromatic region of the ^1^H NMR spectrum of **S3** (500 MHz, [D]chloroform,300 K). | **S8** |
| **Figure** **S3**. The crown ether region of the ^1^H NMR spectrum of **S3** (500 MHz, [D]chloroform, 300 K). | **S9** |
| Synthesis of Meso-crowned porphyrin [12]-C-4POR | **S10** |
| **Figure S4**. The ^1^H NMR spectrum of **[12]-C-4POR** (500 MHz, [D]chloroform, 300 K). | **S11** |
| **Figure S5**. The aromatic region of a ^1^H NMR spectrum of **[12]-C-4POR** (500 MHz, [D]chloroform, 300 K). | **S12** |
| **Figure S6**. The crown ether region of the ^1^H NMR spectrum of **[12]-C-4POR** (500 MHz, [D]chloroform, 300 K). | **S12** |
| **Figure S7.** The ^13^C NMR spectrum of **[12]-C-4POR** (125 MHz, [D]chloroform, 125 K). | **S13** |
| **Figure S8**. ^1^H-^1^H COSY NMR spectrum of **[12]-C-4POR** (500 MHz, [D]chloroform, 300 K). | **S13** |
| **Figure S9**. ^1^H-^1^H NOESY NMR spectrum of **[12]-C-4POR** (500 MHz, [D]chloroform, 300 K). | **S14** |
| **Figure S10**. ^1^H-^13^C HSQC NMR spectrum of **[12]-C-4POR** (500 MHz, [D]chloroform, 300 K). | **S14** |
| **Figure S11**. ^1^H-^13^C HMBC NMR spectrum of **[12]-C-4POR** (500 MHz, [D]chloroform, 300 K). | **S15** |
| **Figure S12.** The high-resolution mass spectrum of [12]-C-4POR–Pb (ESI+, TOF). | **S15** |
| **Figure S13.** The high-resolution mass spectrum of [12]-C-4POR–Pb (ESI+, TOF). Top: experimental spectrum, bottom: simulated isotopic pattern | **S16** |
| The synthesis of [12]-C-4POR–Pb | **S17** |
| **Figure S14.** The ^1^H NMR spectrum of **[12]-C-4POR–Pb** (500 MHz, D]chloroform, 300 K). | **S18** |
| **Figure S15**. The aromatic region of the ^1^H NMR spectrum of **[12]-C-4POR–Pb** (500 MHz, [D]chloroform, 300 K). | **S18** |
| **Figure S16.** The crown ether region of the ^1^H NMR spectrum of **[12]-C-4POR–Pb** (500 MHz, [D]chloroform, 300 K). | **S19** |
| **Figure S17.** The ^13^C NMR spectrum of **[12]-C-4POR–Pb** (125 MHz,D]chloroform, 300 K). Signals corresponding to **[12]-C-4POR** were marked with asterisks. Demetallation was observed during the measurements, explaining the appearance of signals corresponding to the free ligand. | **S19** |
| **Figure S18.** The high-resolution mass spectrum of **[12]-C-4POR–Pb** (ESI+, TOF). | **S20** |
| **Figure S19.** The high-resolution mass spectrum of **[12]-C-4POR–Pb** (ESI+, TOF). Top: experimental spectrum, bottom: simulated isotopic pattern. | **S20** |
| **Figure S20.** The ^1^H NMR spectra (600 MHz, [D_3_]acetonitrile, 300 K) of **[12]-C-4POR** before and after the addition of lithium bis(trifluoromethanesulfonyl)imide (>100 equiv.). | **S21** |
| **Figure S21.** The high-resolution mass spectrum of **[12]-C-4POR–Pb** upon the addition of Li^+^ (ESI+, TOF). | **S21** |
| **Figure S22.** The XPS spectra of control and passivated films. | **S21** |
| **Figure S23.** XPS spectra of the control and [12]-C-4POR powder and [12]-C-4POR-based perovskite films (a) Iodide (I3d5/2) (b) Oxygen (O1s) (c) N1S | **S22** |
| **Figure S24.** The high-resolution mass spectrum of the solution of **[12]-C-4POR** and lithium trifluoromethanesulfonate in DMSO/MeCN/DCM mixture (8:1:1) (ESI+, TOF). | **S22** |
| **Figure S25.** 3D AFM images of the control and **[12]-C-4POR**-based perovskite films. | **S23** |
| **Figure S26.** KPFM images of the (a) control and (b) **[12]-C-4POR**-based perovskite films. | **S23** |
| **Figure S27.** Current density−voltage (J−V) curves of control and [12]-C-4POR at different concentrations for the devices | **S23** |
| **Figure S28.** Statistics of the photovoltaic parameter distributions of 10 independent devices under both forward and reverse scan at different concentrations | **S24** |
| **Figure S29.** Nyquist plots of impedance spectra for the (a) control and (b) **[12]-C-4POR**-incorporated devices under a 0.6 and 0.8 V bias. | **S24** |
| **Figure S30.** The current–voltage curves of hole-only devices employing control and passivated perovskite films | **S25** |
| **Figure S31.** Current density–voltage (J–V) characteristics of the [12]-C-4POR and control devices measured after 15 days of shelf-life stability. | **S25** |
| **Table S1.** Summary of XPS analysis, peak position, full width at half maximum (FWHM), and atomic concentration (%At) of detected elements for the control film. | **S26** |
| **Table S2.** Summary of XPS analysis, peak position, full width at half maximum (FWHM), and atomic concentration (%At) of detected elements for the [12]-C-4POR-based perovskite films. | **S26** |
| **Table S3**. Summary of XPS analysis, peak position, full width at half maximum (FWHM), and atomic concentration (%At) of detected elements for the [12]-C-4POR powder. | **S27** |
| **Table S4.** Carrier lifetimes of perovskite films, pristine and passivated with [12]-C-4POR. | **S27** |
| **Table S5**. Performance of PSCs with different [12]-C-4POR concentrations. | **S27** |
| **Table S6.** Summary of the Photovoltaic Parameters for the Best Control and [12]-C-4POR Modulated PSCs. | **S28** |
| **Table S7.** Previously reported crown ether or porphyrin-based molecules for efficient and stable perovskite solar cells. | **S29** |
| **Table S8.** The EIS parameters of bare and [12]-C-4POR passivated perovskite. | **S30** |
| **References** | **S30** |

**Experimental procedures**

**Materials.** Unless indicated, all compounds and solvents were utilized without additional purification. Chempur (Poland) provided the solvents. The following materials were purchased from Sigma-Aldrich: lead iodide (PbI_2_, 99.9985%, trace metal basis), lead bromide (PbBr_2_) (98%), spiro-OMeTAD (99%), bis(trifluoromethane)sulfonimide lithium salt (LiTFSI) (99.95%), 4-tert-butylpyridine (tBP), [N,N-dimethylformamide (DMF) (anhydrous, 99.8%), dimethyl sulfoxide (DMSO) (anhydrous, 99.8%), chlorobenzene (CB) (anhydrous, 99.8%), and acetonitrile (anhydrous, 99.8%). Greatcell Solar Ltd. supplied the formamidinium iodide (>99.99%), methylammonium bromide (>99.99%), and FK 209 Co (III)TFSI (98%). Alfa Aesar supplied the tin (IV) oxide (SnO_2_) aqueous colloidal solution (15% in H_2_O colloidal dispersion), which contains SnO_2_ colloidal particles.

**Device Fabrication.** Fluorine-doped tin oxide (FTO) glass substrates were cleaned for 15 minutes using Helmanex, DI water, acetone, and isopropyl alcohol, respectively, and then exposed to UV ozone for 20 minutes. A compact SnO_2_ layer approximately 20–30 nm thick was subsequently deposited onto the FTO surface using spin coating at 4000 rpm for 30 seconds, followed by 30 minutes of annealing at 150 °C. A triple cation perovskite solution of 1.5 M with the composition [Cs_0.05_(FA_0.90_MA_0.10_)_0.95_Pb(I_0.90_Br_0.10_)_3_] was prepared in DMSO: DMF = 4 : 1 solvents. Spin-coating the perovskite solution included 10 s at 1000 rpm and 30 s at 6000 rpm. In the second step, chlorobenzene was introduced as an anti-solvent onto the spinning perovskite surface 15 seconds before the end of the process. Subsequently, each substrate was promptly transferred to the hot plate and allowed to anneal for one hour at 100 °C. Then, the **[12]-C-4POR** was dissolved in chlorobenzene and spin-coated for 20 s at 4000 rpm. Subsequently, spiro-OMeTAD solution (70 mM) in 1 mL of chlorobenzene containing tBP, Li-TFSI and FK209 solution in a molar ratio of spiro-OMeTAD/FK209/Li-TFSI/TBP of 1:0.03:0.5:3.3 was spin-coated for 20 s at 4000 rpm. Finally, the 80 nm of gold (Au) electrodes were deposited with a thermal evaporator.

**Current Density−Voltage (J−V) Measurements.** The photovoltaic performance was assessed using a Fluxim Litos Lite system, which included a Wavelabs Sinus LS2 solar simulator with an AM 1.5 spectrum for excitation. The current-voltage characteristics were obtained by conducting forward and reverse scans at a scan rate of 50 mV s^−1^ on masking devices having a pixel size of 0.09 cm^2^. A calibrated reference solar cell made of crystalline silicon, along with a KG-5 filter, was used to establish the light intensity at 100 mW cm^−2^. The power output was stabilized by using an MPP tracking algorithm for 300 seconds. Operational stability of the control and **[12]-C-4POR** based devices under MPP tracking with continuous 1 sun illumination under a N_2_ atmosphere. Shelf‐life stability of the control and **[12]‐C‐4POR**‐based devices under daylight with RH ∼55% was carried out. Thermal stability of the control and **[12]‐C‐4POR**‐based devices at 65°C under N_2_ atmosphere was recorded.

**X-Ray Photoelectron Spectroscopy (XPS)** was performed using the ULVAC-PHI VersaProbe 5000 spectrometer, which used monochromatic Al Ka radiation with a photon energy of 1486.6 electron volts (eV).

**Scanning Electron Microscopy (SEM).** The field emission scanning electron microscopy (FE-SEM) technique (S5500, Hitachi) was used to evaluate the top-view and cross-section SEM of all films.

**Contact angle measurements** were conducted using a Biolin Scientific Attention – Theta Lite contact-angle device at room temperature, using water as the test liquid.

**Time-Resolved Photoluminescence (TRPL)** were obtained by a fluorescence spectrometer equipped with a 405 nm pulsed laser (Edinburgh Instruments FS5 model Spectrometer).

**External Quantum Efficiency (EQE)** was obtained at room temperature using a QE system (EnliTech) with monochromatic light focused on one pixel of the device with a cutoff frequency of 20 Hz.

**Electrochemical Impedance Spectroscopy (EIS)** measurements of all PSCs were performed on a self-designed sample holder using Ivium potentiostat equipped with a frequency response analyzer under dark as a function of applied bias.

**Atomic Force Microscopy (AFM).** Surface roughness values and surface topography of the films were obtained with an atomic force microscope (AFM) (Park System XE-100E).

**NMR spectroscopy.** The ^1^H NMR and ^13^C NMR spectra were recorded on high-field Bruker spectrometers (600 MHz and 500 MHz) equipped with a broadband inverse gradient probe head. The spectra were referenced to the residual solvent signal ([D]chloroform, 7.24 ppm; [D_2_]dichloromethane, 5.32 ppm; [D_3_]acetonitrile, 1.94 ppm).

**Mass spectrometry**. ESI mass spectra were recorded on a Bruker qTOF compact.

**Secondary ion mass spectrometry** depth profiling experiment was conducted using the TOF.SIMS 5 system (ION-TOF GmbH, Germany), equipped with a time-of-flight mass analyzer (TOF-SIMS). Samples were analyzed without any special pretreatment upon receipt, and they were transferred directly to the analysis chamber, where the pressure was maintained at 9 × 10^−10^ mbar. Elemental distributions were acquired using the dual beam mode in noninterlaced mode. The degree of ion distribution in the sample was obtained by sputtering of oxygen ions (operating conditions: 1000 eV, 240-250 nA ion current) over a 250 μm × 250 μm area for positive ions analysis or cesium ions (operating conditions: 1000 eV, 62-65 nA ion current) for negative ions analysis. Subsequently, the internal layers of the sample were analyzed using a Bi^3+^ ion beam (operating conditions: 64 μm × 64 μm raster size, 30 keV, 0.48 pA ion current). Internal mass calibration was performed using the mass of ions that are consistently present: a series of CxHy+ or CxHy- ions depending on the polarity mode.

**Scheme S1.** The synthesis of porphyrin **[12]-C-4POR.**

**S3** was obtained under modified literature conditions.^[1]^

In a dry two-necked round-bottom flask, dry acetonitrile (100 ml) was introduced and purged with argon for 15 minutes. After the addition of 3,4-dihydroxybenzaldehyde **S1** (0.9 g, 6.51 mmol), purging was continued for the next 15 minutes. Caesium carbonate (5.5 g, 16.7 mmol) was added, and the heterogeneous mixture was refluxed for 3 hours. After that time, the solution turned a bright yellow color. The solution of triethylene glycol di(*p*-toluenesulfonate) **S2** (3.07 g, 6.7 mmol) in acetonitrile (50 mL) was introduced dropwise into the refluxing heterogeneous mixture using a syringe pump over a period of 12 hours. After complete addition, the reaction mixture was refluxed for 24 hours. The reaction mixture was cooled to room temperature and filtered through filter paper. The filtrate was evaporated in vacuo, and the residue was dissolved in dichloromethane (100 mL). The organic phase was washed with 1 M HCl (50 mL), H_2_O (100 mL), and brine (100 mL), then dried over anhydrous Na_2_SO_4_ and evaporated under reduced pressure to obtain the crude product. The latter was purified by column chromatography (ethyl acetate, dichloromethane, and hexane eluent with a proportion of 3:1:1) to provide aldehyde **S3** (435 mg, 1.73 mmol; 26%) as a white solid.

**^1^H NMR** (500 MHz, [D]chloroform, 300 K): δ (ppm) 9.78 (s, 1H, H_a_), 7.47–7.43 (m, 1H, H_c_), 7.46 (s, 1H, H_b_), 6.98 (d, 1H, H ^3^*J* = 8.3 Hz, H_d_), 4.20–4.15 (m, 4H, H_crown_), 3.84–3.81 (m, 2H, H_crown_), 3.78–3.74 (m, 2H, H_crown_) 3.70 (s, 4H, H_crown_).

The analytical data are in agreement with the published data.^[1]^

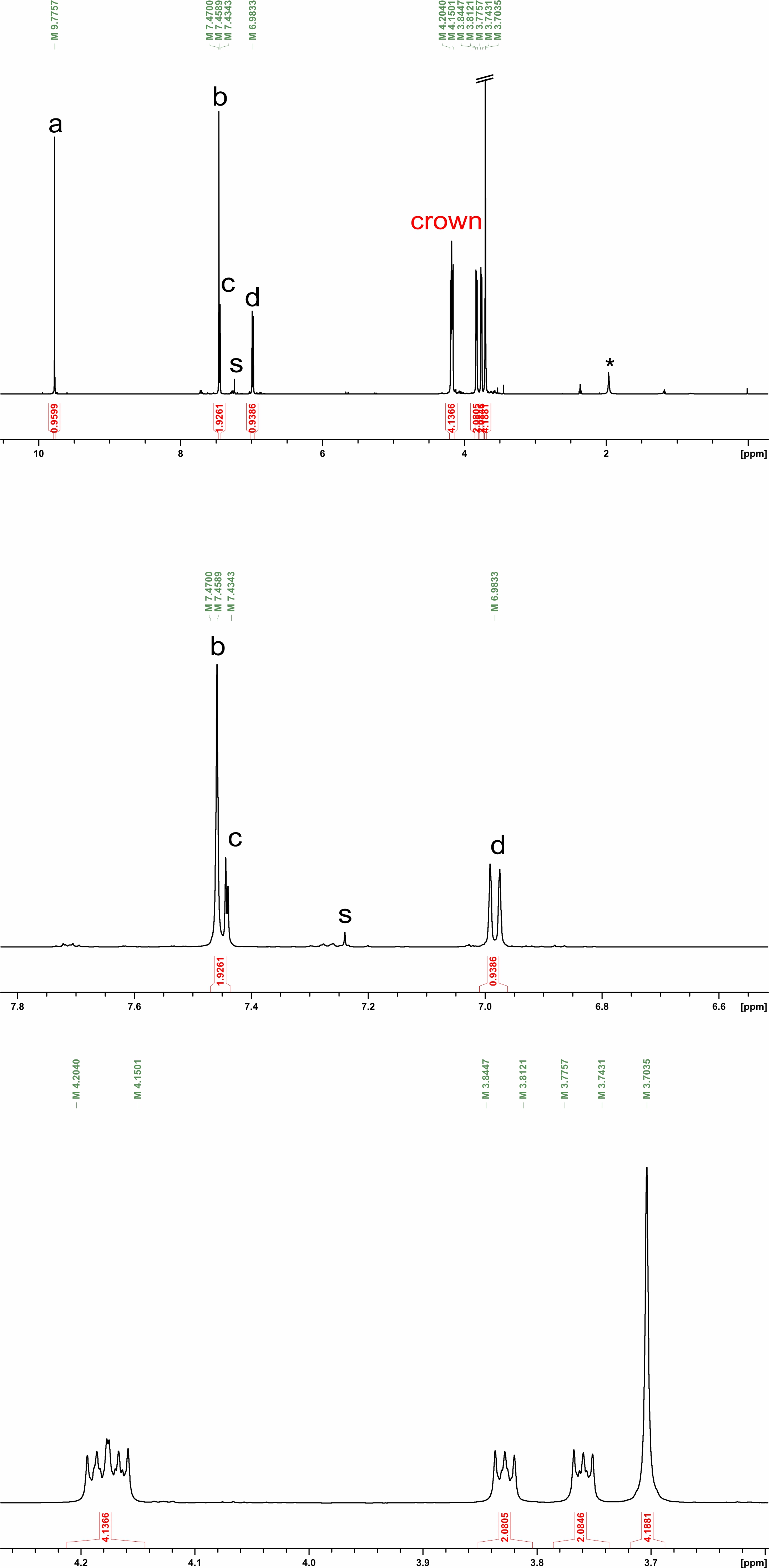


**Figure S1.** The ^1^H NMR spectrum of **S3** (500 MHz, [D]chloroform, 300 K).


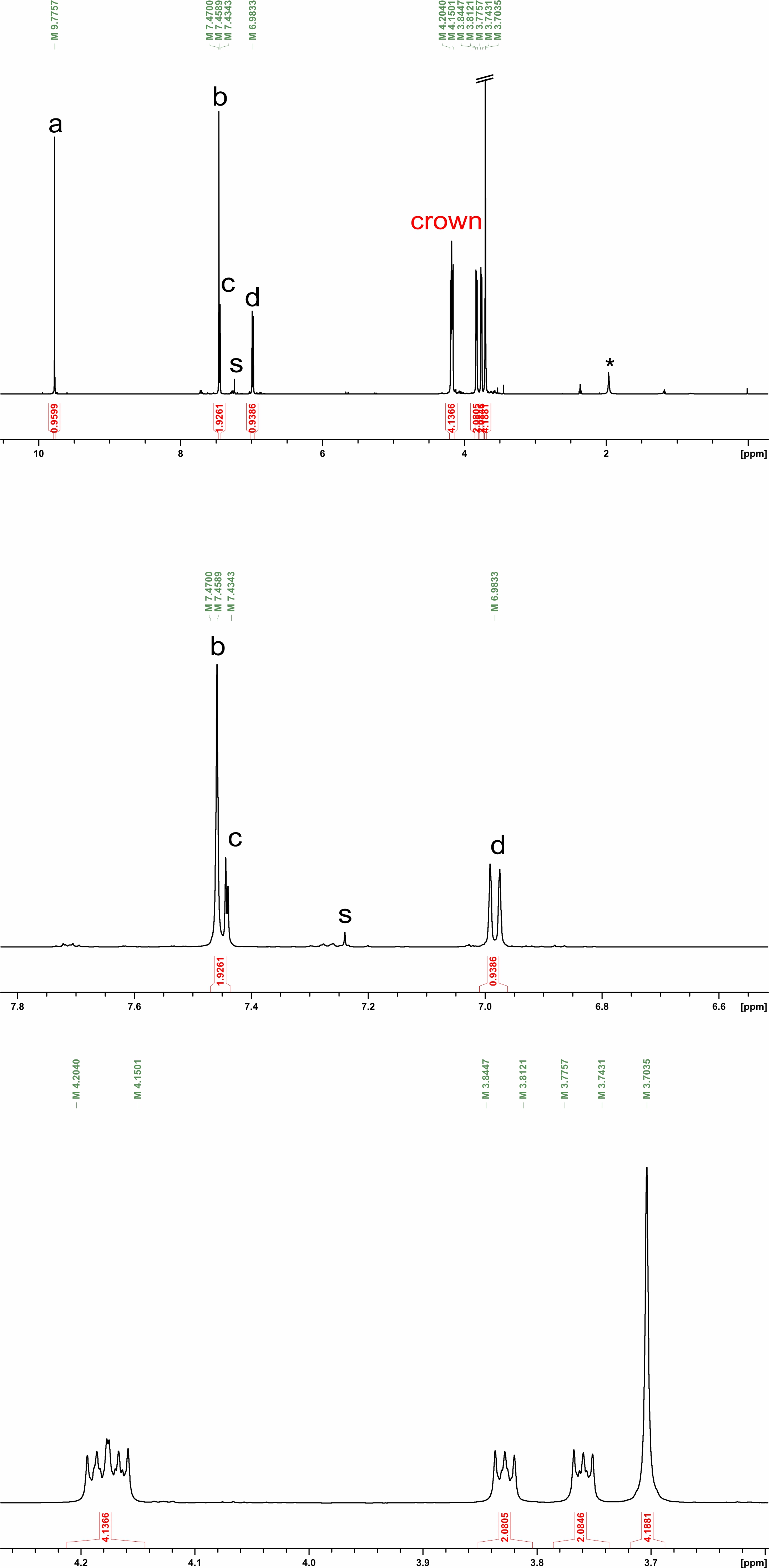


**Figure S2.** The aromatic region of the ^1^H NMR spectrum of **S3** (500 MHz, [D]chloroform,
300 K).


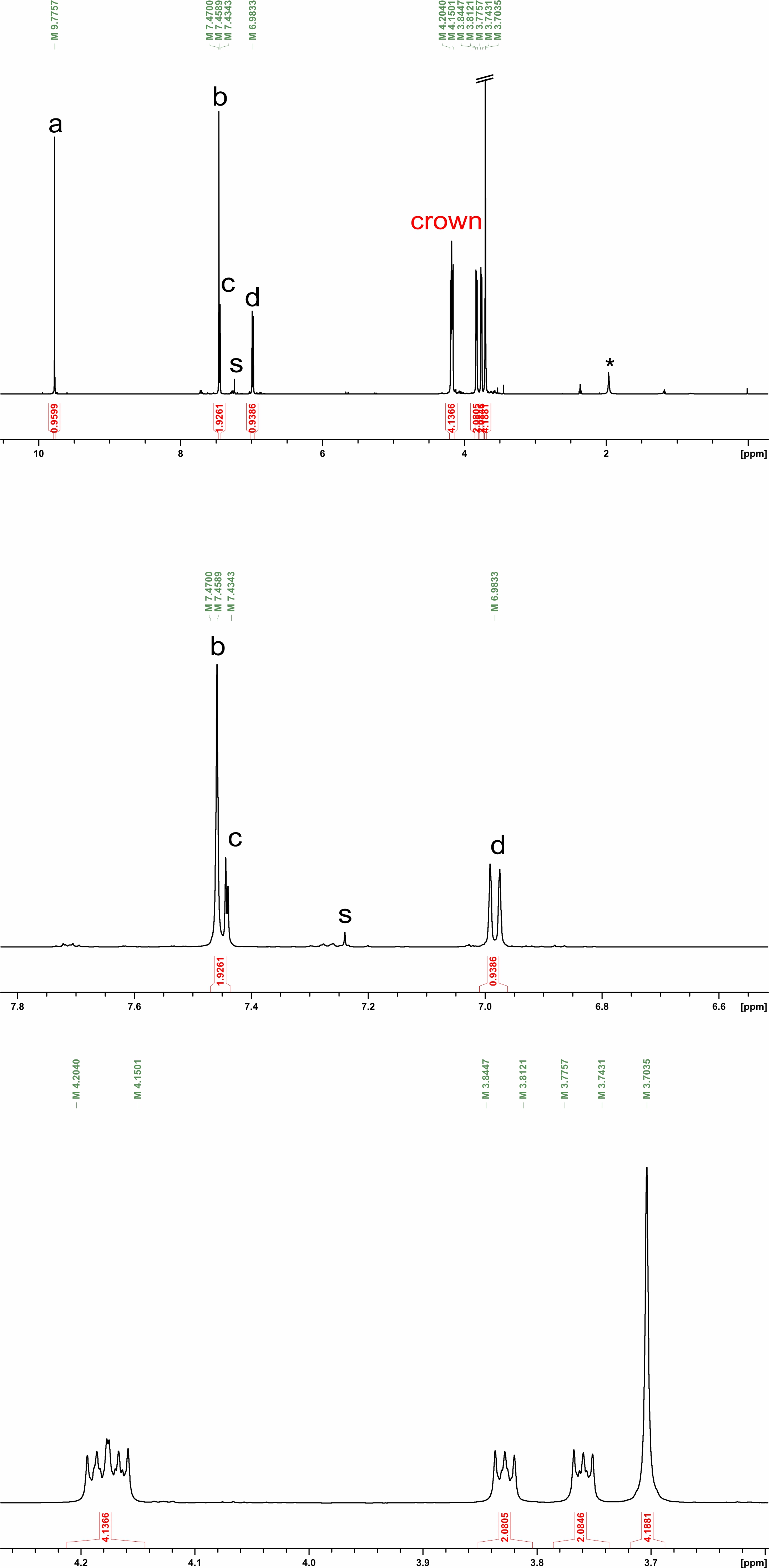


**Figure S3.** The crown ether region of the ^1^H NMR spectrum of **S3** (500 MHz, [D]chloroform, 300 K).

***Meso*-crowned porphyrin [12]-C-4POR**

In a 25 ml round-bottom flask, **S3** (354 mg, 1.4 mmol), pyrrole (100 μL, 1.4 mmol), and propionic acid (8.5 mL) were introduced. The pyrrole was previously purified by passing it through a column filled with aluminum oxide. The reaction was carried out under reflux for 30 minutes. After that time, the reaction mixture was partitioned between dichloromethane (dichloromethane, 50 mL) and distilled water (50 mL) and transferred to a separatory funnel. The reaction mixture was neutralised with saturated NaHCO_3_ solution in distilled water. The organic fraction was separated, and the water fraction was extracted one more time with dichloromethane (50 mL). The combined organic phases were washed with brine (50 mL) and then dried over anhydrous sodium sulfate (Na_2_SO_~~4~~_). The drying agent was removed *via* gravity filtration, and the filtrate was evaporated to dryness. The product was purified *via* flash chromatography (dichloromethane with 0-4 % methanol gradient) to provide **[12]-C-4POR** (61.5 mg, 51.3 μmol, 15% yield) as a violet solid.

**[12]-C-4POR**

**^1^H NMR** (500 MHz, [D]chloroform, 300 K): δ (ppm) 8.87 (s, 8H, H_b_), 7.82 (d, 4H, ^4^*J* = 2.1 Hz, H_e_), 7.77 (dd, 4H, ^3^*J* = 8.1 Hz, ^4^*J* = 2.1 Hz, H_c_), 7.32 (d, 4H, ^3^*J* = 8.1 Hz, H_d_), 4.51−4.46 (m, 8H, H_k_), 4.36−4.32 (m, 8H, H_f_), 4.08−4.04 (m, 8H, H_j_), 3.97–3.91 (overlapping signals, 24 H, H_g_, H_h_, H_i_), −2.82 (s, 2H, H_a_).

**^13^C NMR** (125 MHz, [D]chloroform, 300 K): δ (ppm) 150.4, 148.8, 136.6, 129.2, 124.6, 119.6, 116.2, 72.2, 72.0, 71.4 (two overlapping signals), 70.1, 70.0. *Some of the signals could not be identified because of the broadening of the lines.*

**HRMS** (ESI+, TOF): *m/z* [M+H]^+^: 1199.4705, calcd. for C_68_H_71_N_4_O_16_^+^: 1199.4860.


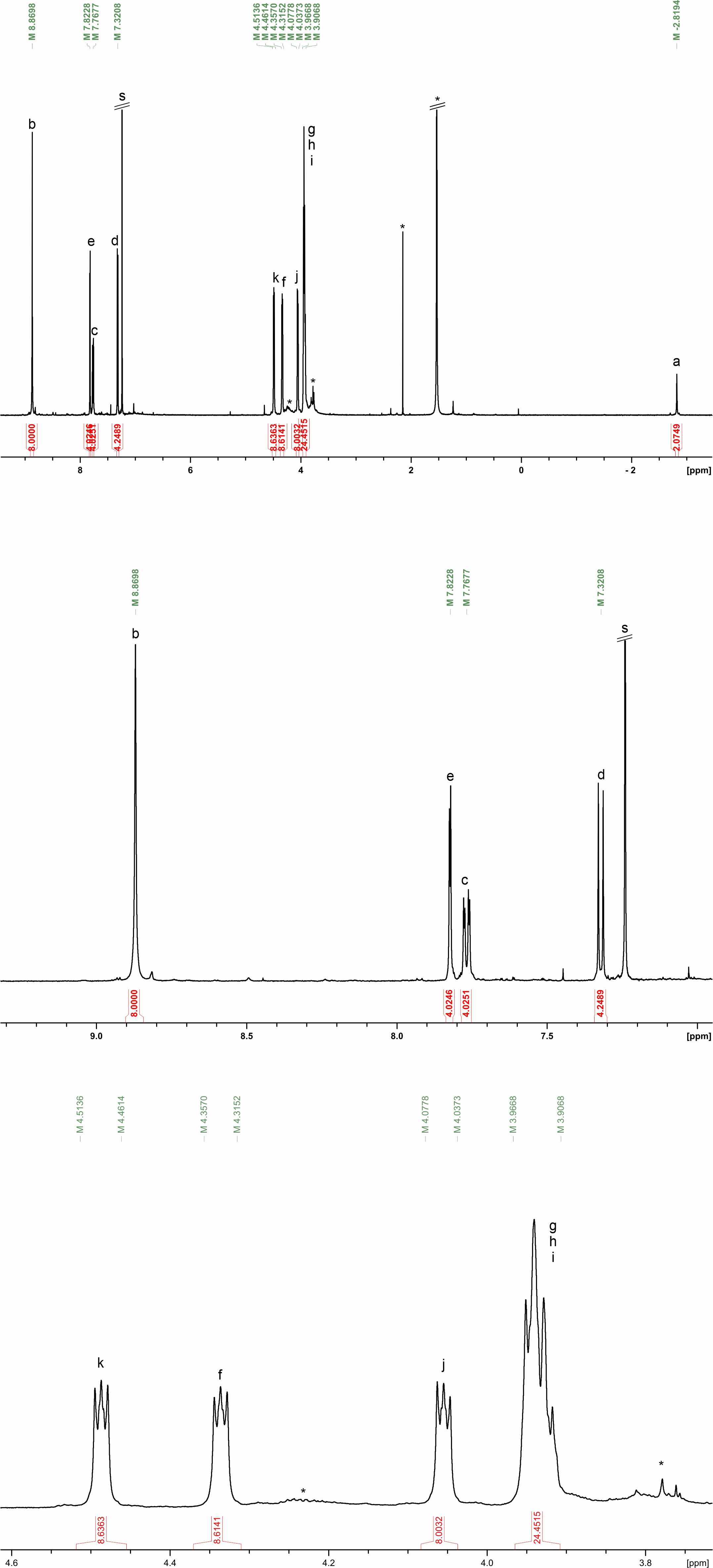


**Figure S4.** The ^1^H NMR spectrum of **[12]-C-4POR** (500 MHz, [D]chloroform, 300 K).


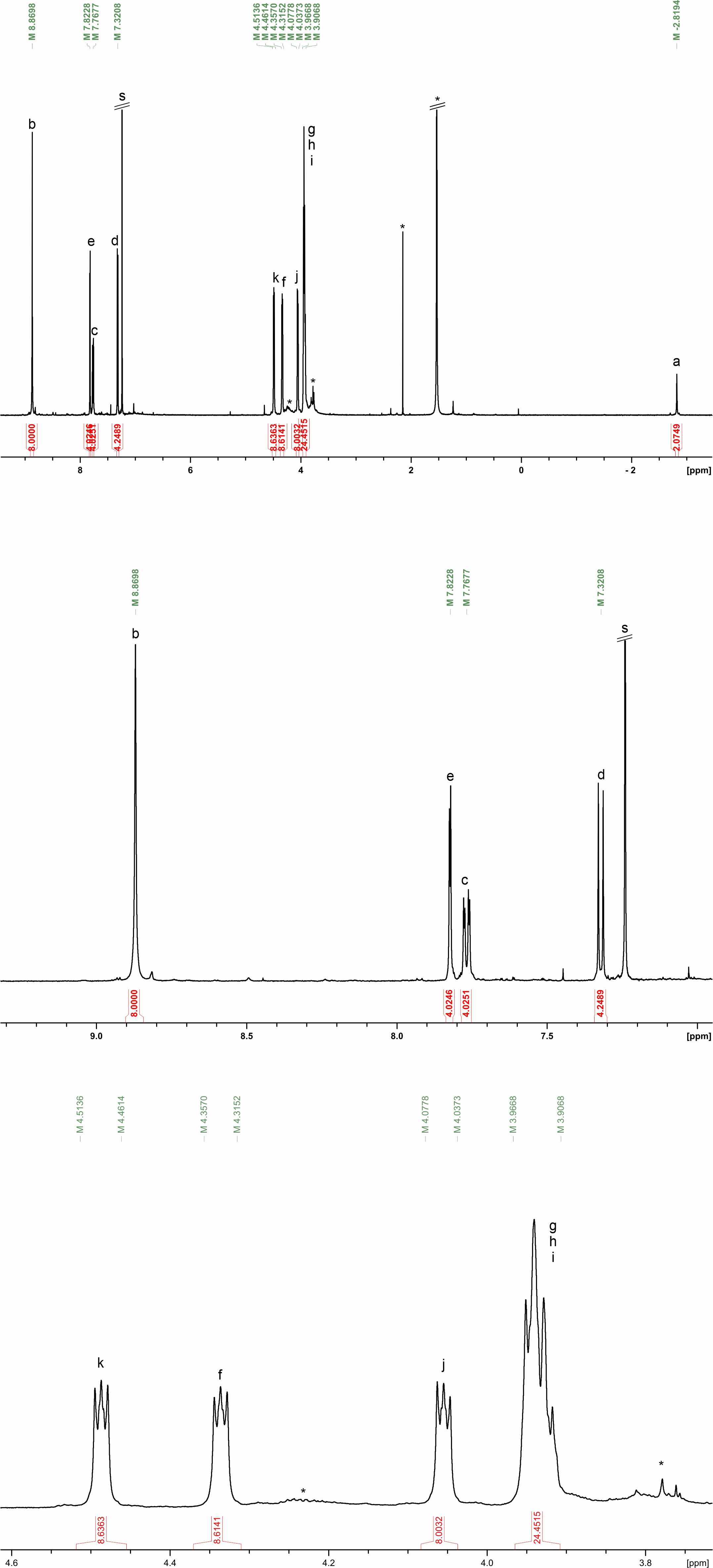


**Figure S5.** The aromatic region of a ^1^H NMR spectrum of **[12]-C-4POR** (500 MHz, [D]chloroform, 300 K).


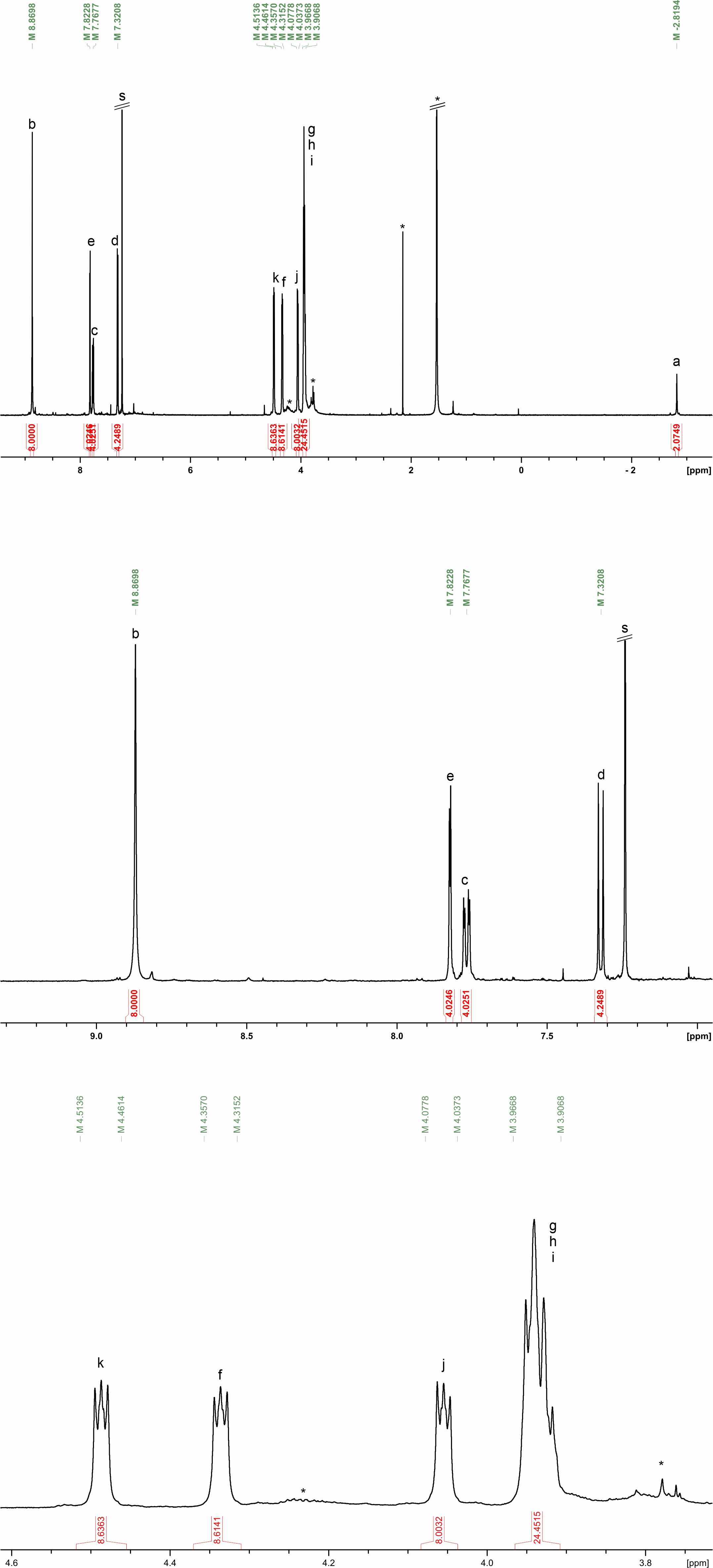


**Figure S6.** The crown ether region of the ^1^H NMR spectrum of **[12]-C-4POR** (500 MHz, [D]chloroform, 300 K).


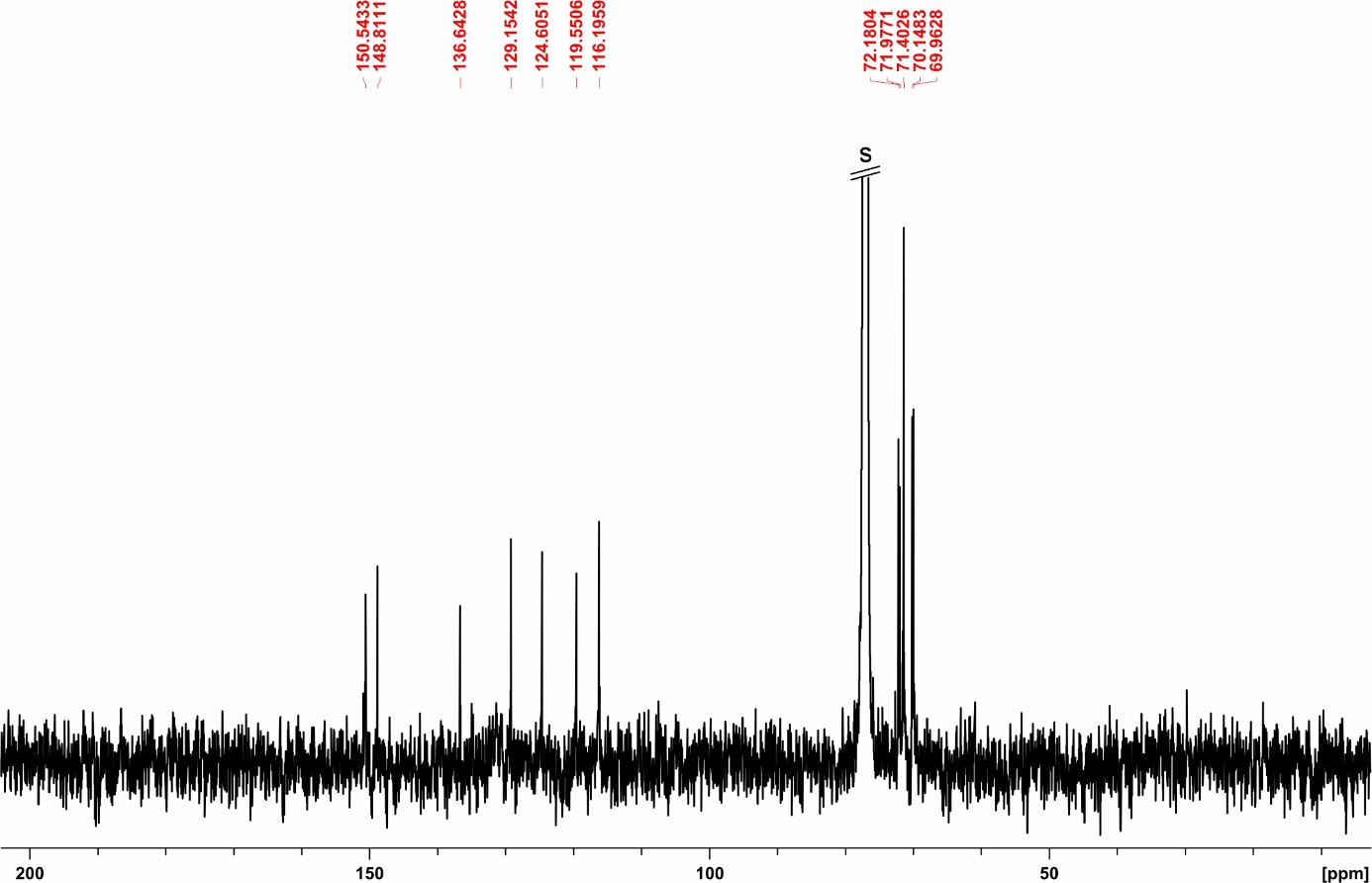


**Figure S7.** The ^13^C NMR spectrum of **[12]-C-4POR** (125 MHz, [D]chloroform, 125 K).


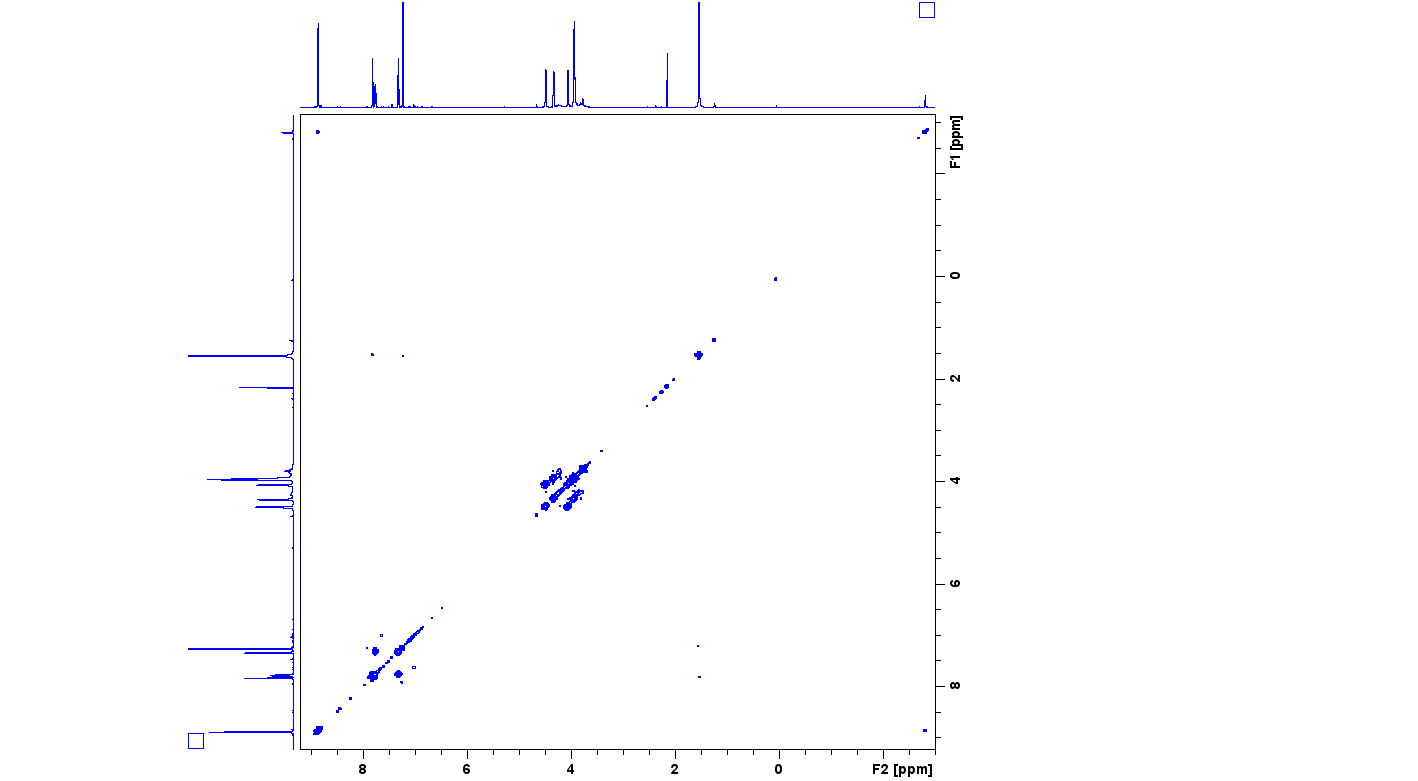


**Figure S8.** ^1^H-^1^H COSY NMR spectrum of **[12]-C-4POR** (500 MHz, [D]chloroform, 300 K).


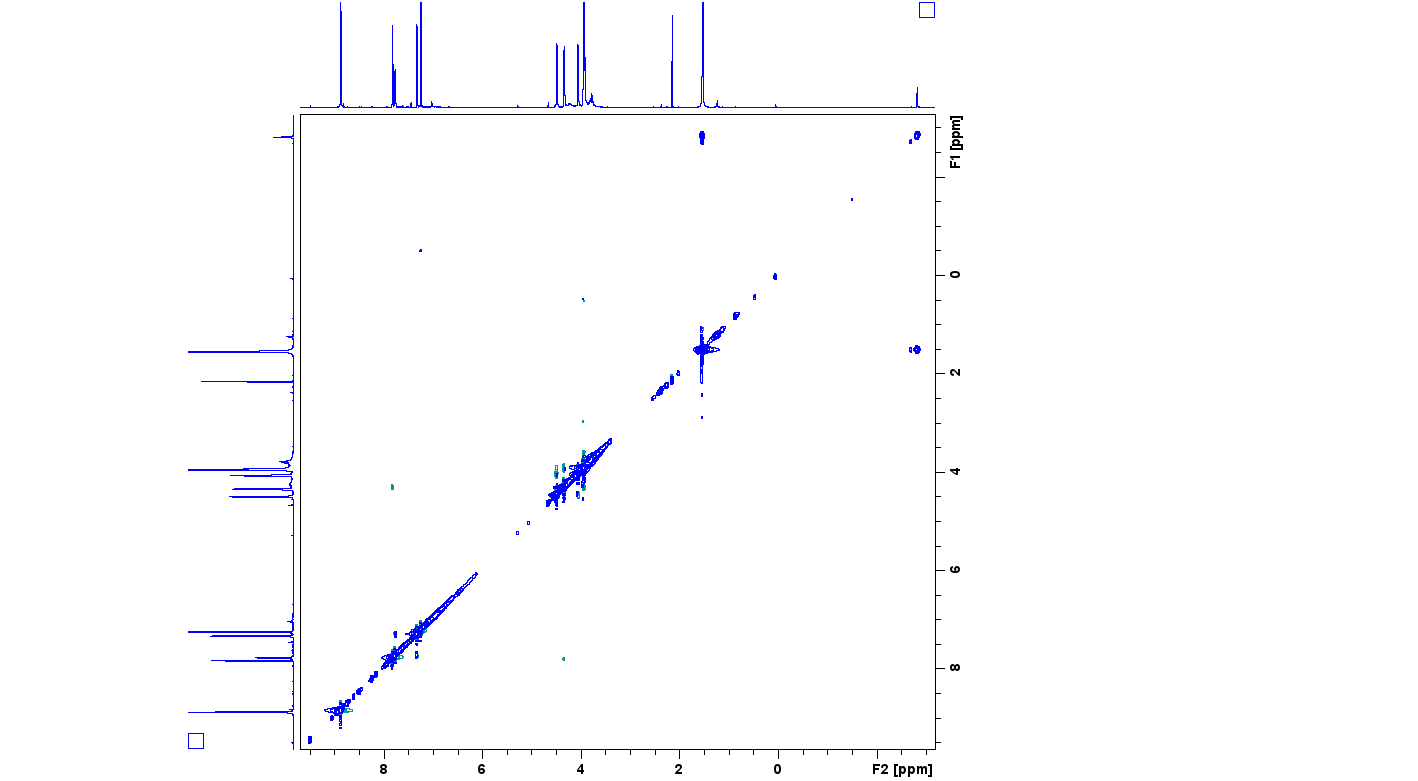


**Figure S9**. ^1^H-^1^H NOESY NMR spectrum of **[12]-C-4POR** (500 MHz, [D]chloroform,
300 K).


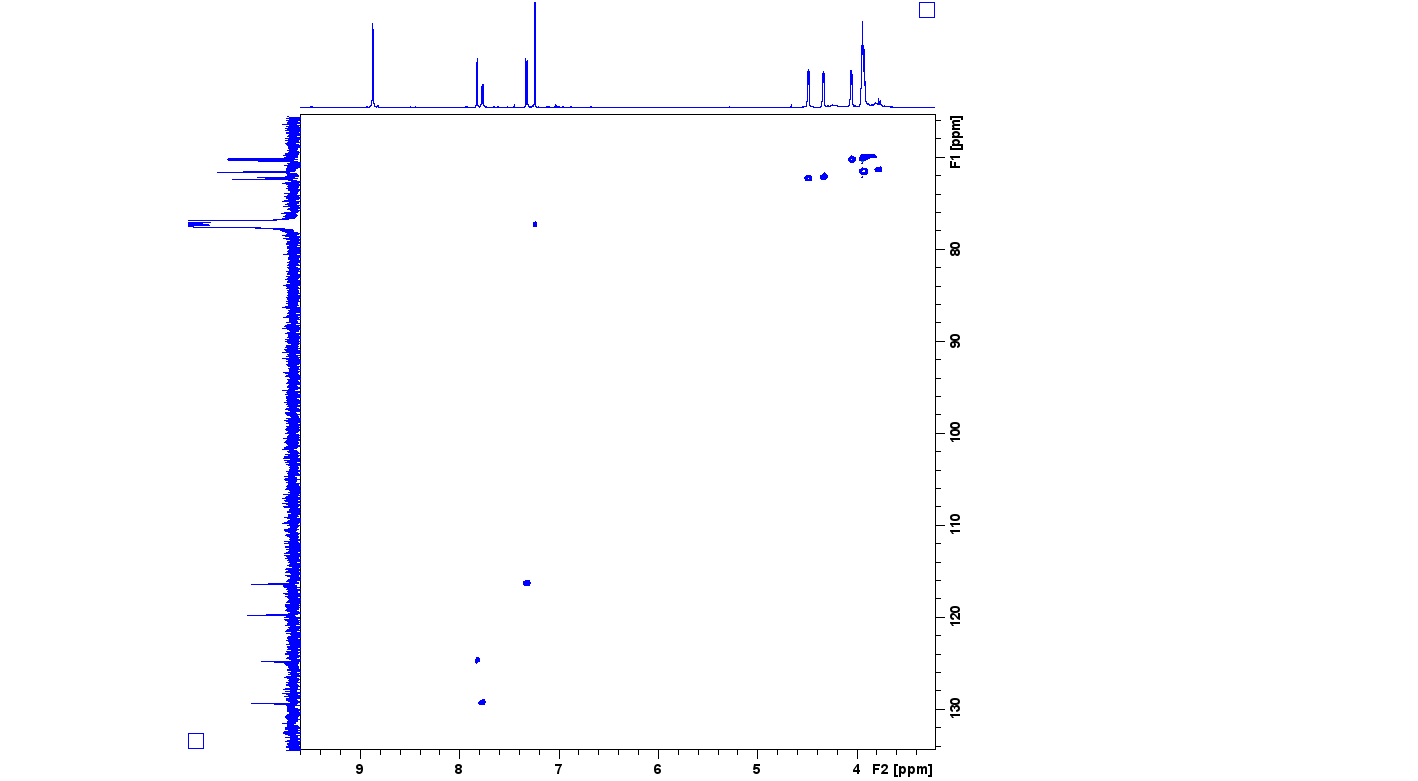


**Figure S10.** ^1^H-^13^C HSQC NMR spectrum of **[12]-C-4POR** (500 MHz, [D]chloroform,
300 K).


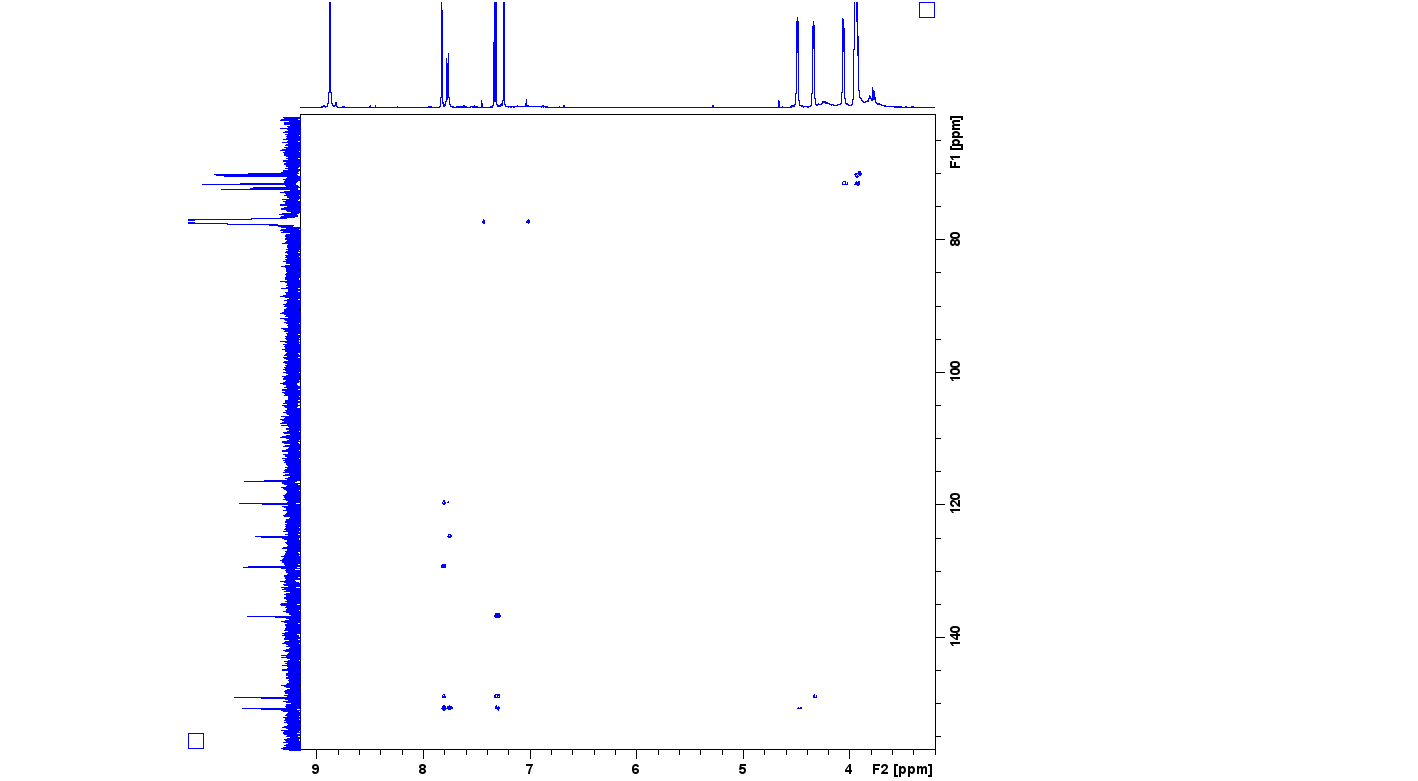


**Figure S11.** ^1^H-^13^C HMBC NMR spectrum of **[12]-C-4POR** (500 MHz, [D]chloroform,
300 K).


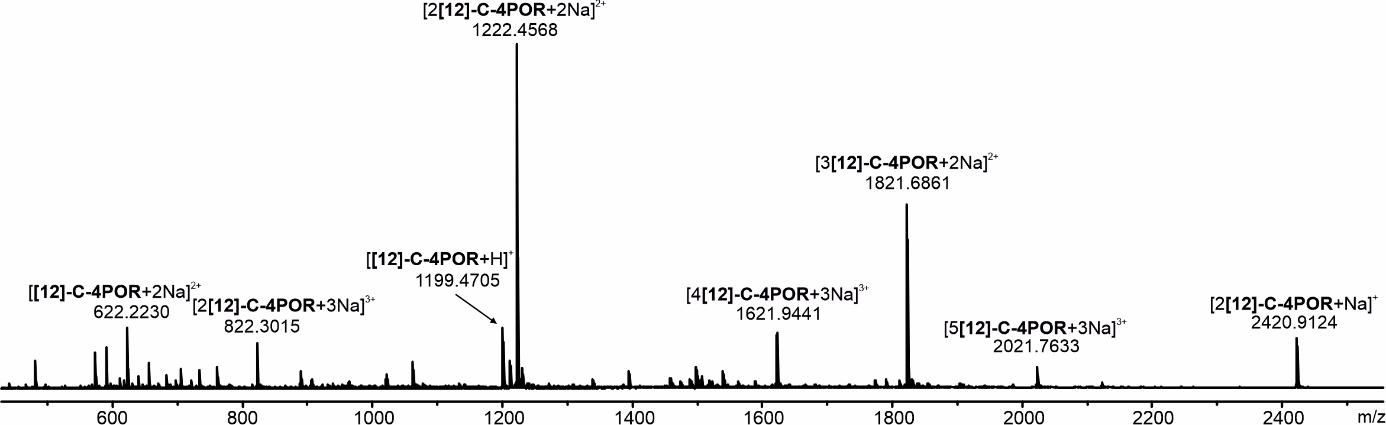


**Figure S12.** The high-resolution mass spectrum of **[12]-C-4POR** (ESI+, TOF).


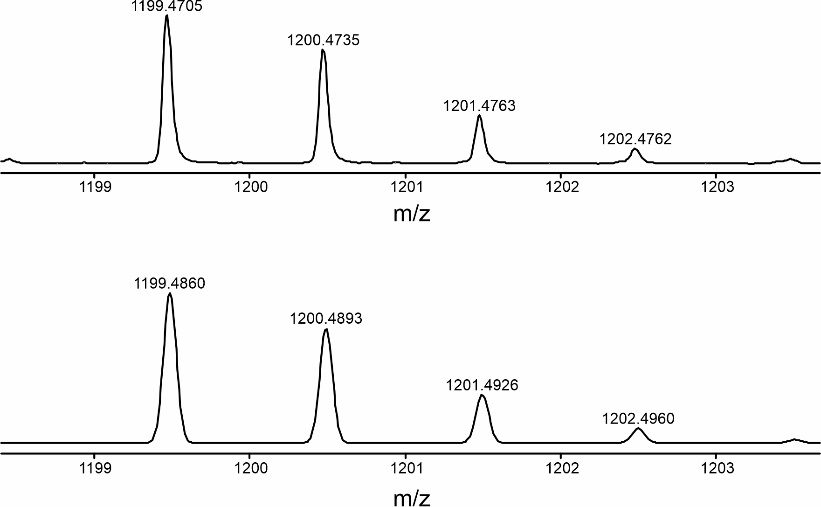


**Figure S13.** The high-resolution mass spectrum of **[12]-C-4POR** (ESI+, TOF). Top: experimental spectrum, bottom: simulated isotopic pattern.

**The synthesis of [12]-C-4POR–Pb**

In a 25 ml round-bottom flask, **[12]-C-4POR** (5.5 mg, 4.6 μmol), Pb(OAc)_2_·3 H_2_O (55 mg, 145 μmol), and methanol (10 mL) were introduced. The reaction was carried out under reflux for 20 hours. After that time, the solvent was evaporated under reduced pressure. The residue was dissolved in dichloromethane and filtered through filter paper. The filtrate was evaporated to dryness to provide **[12]-C-4POR–Pb** as a green solid in a 95:5 proportion to **[12]-C-4POR**. The product decomposed during chromatography and could not be purified by crystallization.

**[12]-C-4POR–Pb**

**^1^H NMR** (500 MHz, [D]chloroform, 300 K, ppm): δ 8.98 (s, 8H, H_β_), 8.07–7.83, 7.76–7.52, 7.40–7.22 (broad, 12H, H*_meso_*_-Ph_), 4.52–4.45, 4.45–4.20, 4.09–4.03, 4.03–3.85 (overlapping, 48H, _Hcrown_).

**^13^C NMR** (125 MHz, [D]chloroform, 300 K, ppm): δ 150.3, 149.4, 148.5, 137.4, 132.0, 121.7, 116.0, 72.1, 72.0, 71.3, 70.1, 69.9. *Some of the signals could not be identified because of the broadening.*

**HRMS** (ESI+, MS): *m/z* [4M+3Pb+3Na]^3+^: 1827.2577, calcd. for C_272_H_274_N_16_O_64_Pb_3_Na_3_^3+^: found 1827.2544.


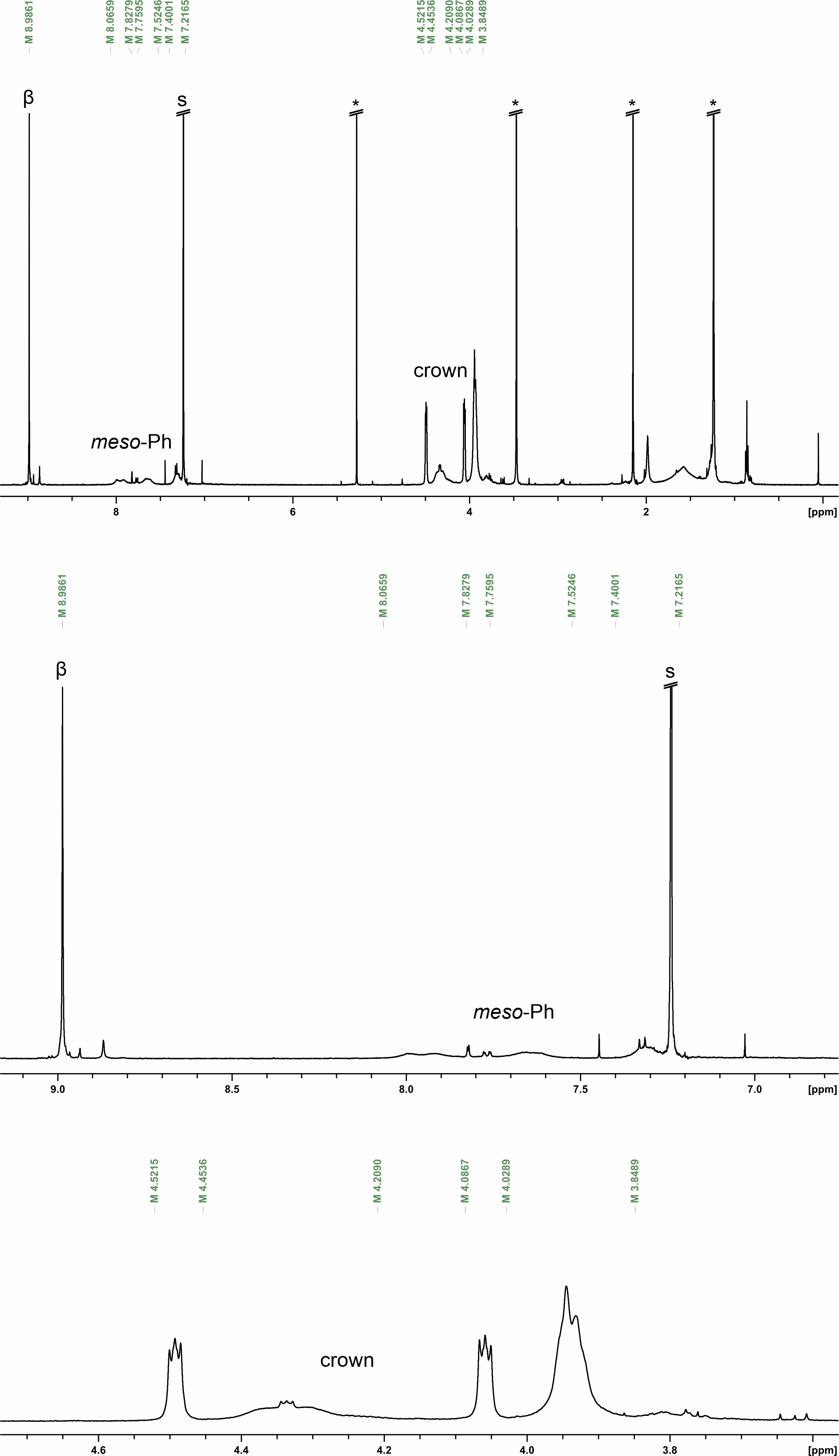


**Figure S14.** The ^1^H NMR spectrum of **[12]-C-4POR–Pb** (500 MHz, [D]chloroform, 300 K).


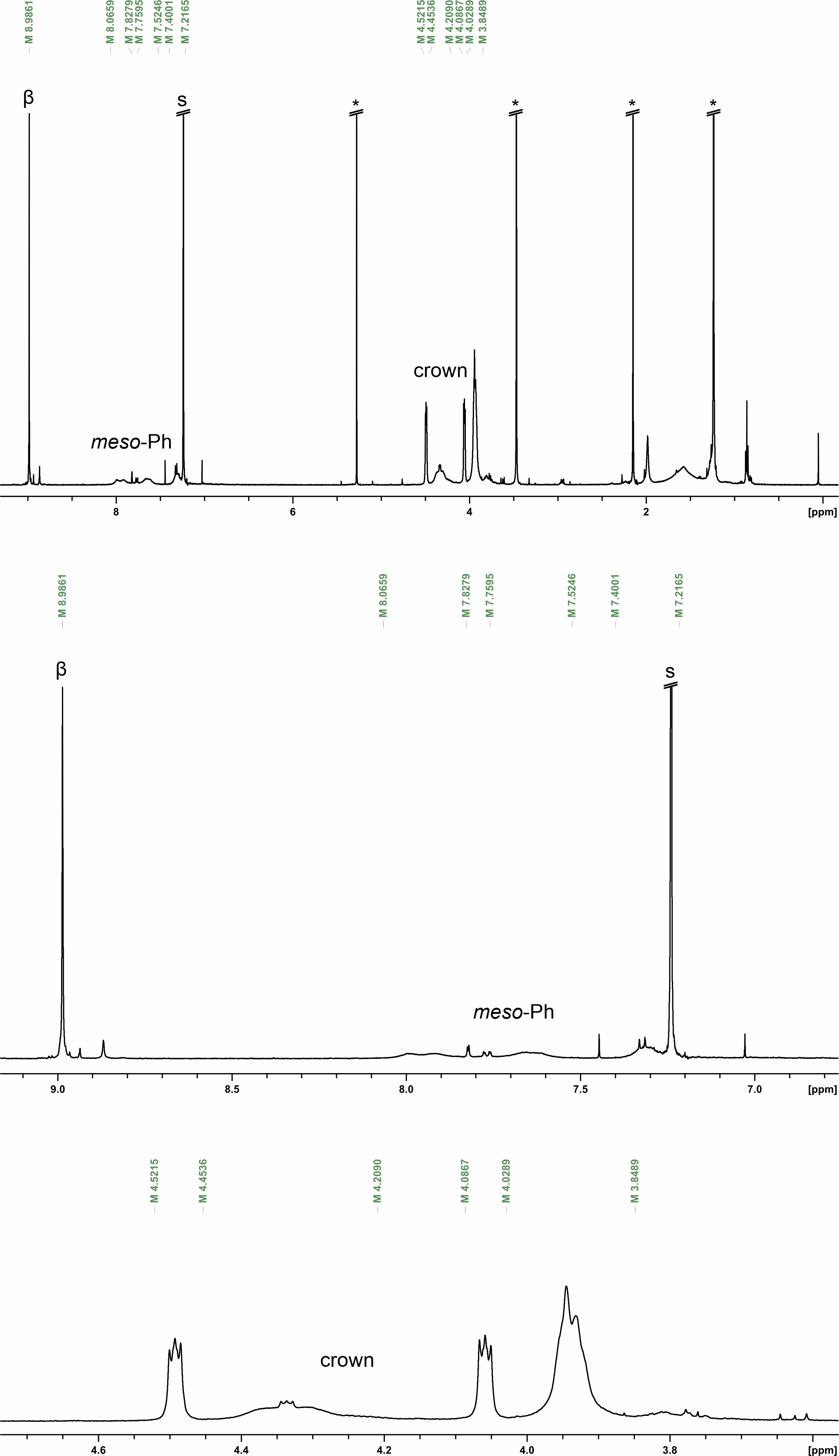


**Figure S15.** The aromatic region of the ^1^H NMR spectrum of **[12]-C-4POR–Pb** (500 MHz, [D]chloroform, 300 K).


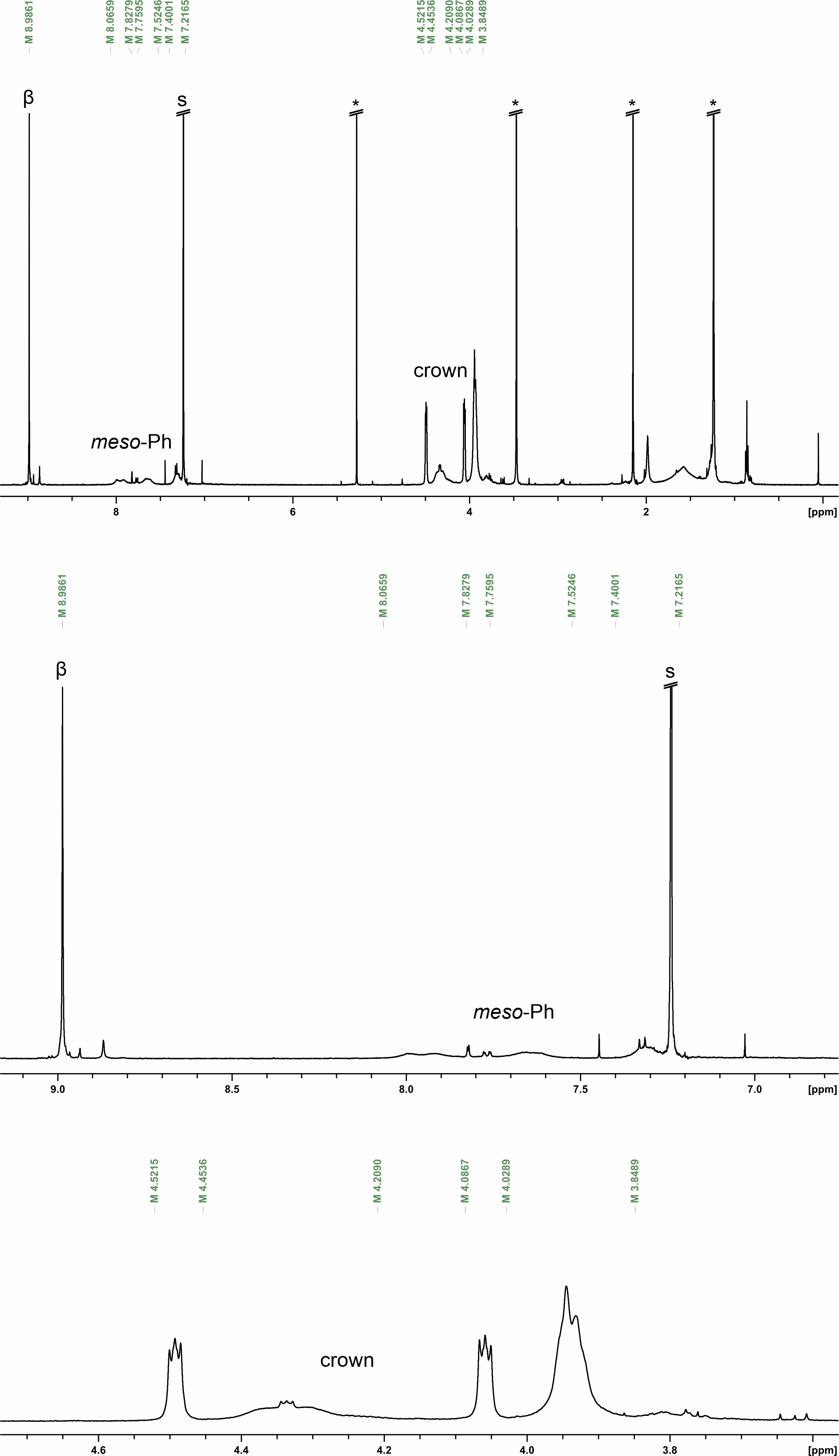


**Figure S16.** The crown ether region of the ^1^H NMR spectrum of **[12]-C-4POR–Pb** (500 MHz, [D]chloroform, 300 K).


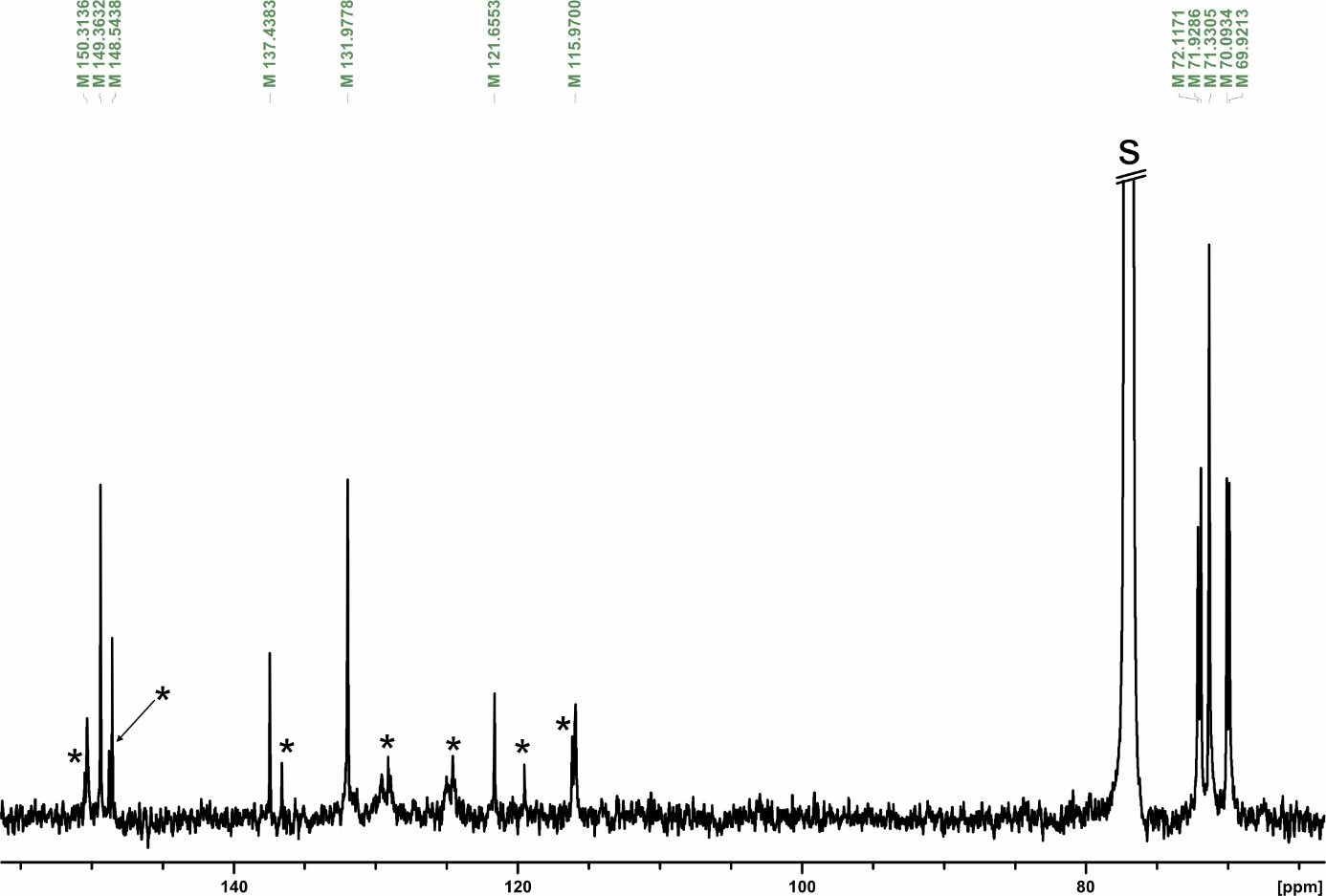


**Figure S17.** The ^13^C NMR spectrum of **[12]-C-4POR–Pb** (125 MHz, [D]chloroform, 300 K). Signals corresponding to **[12]-C-4POR** were marked with asterisks. Demetallation was observed during the measurements, explaining the appearance of signals corresponding to the free ligand.


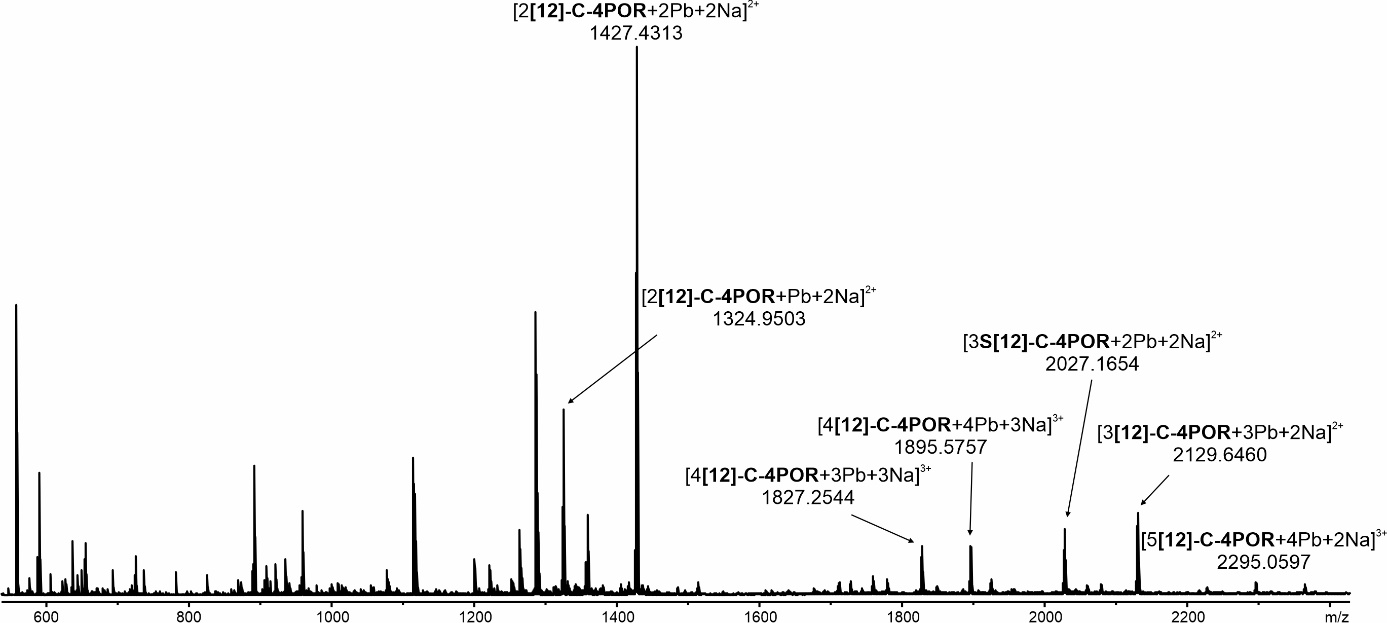


**Figure S18.** The high-resolution mass spectrum of **[12]-C-4POR–Pb** (ESI+, TOF).


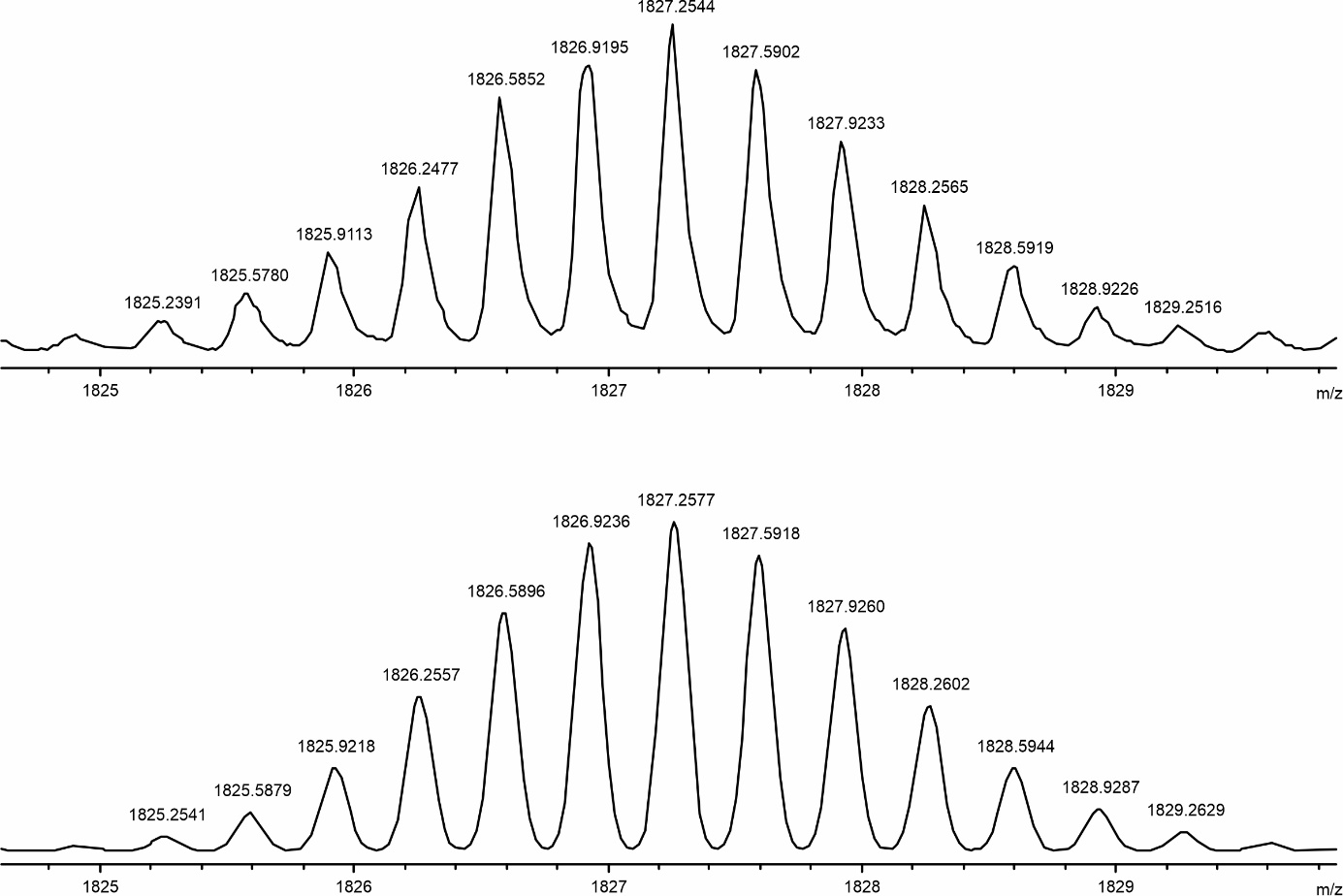


**Figure S19**. The high-resolution mass spectrum of **[12]-C-4POR–Pb** (ESI+, TOF). Top: experimental spectrum, bottom: simulated isotopic pattern.


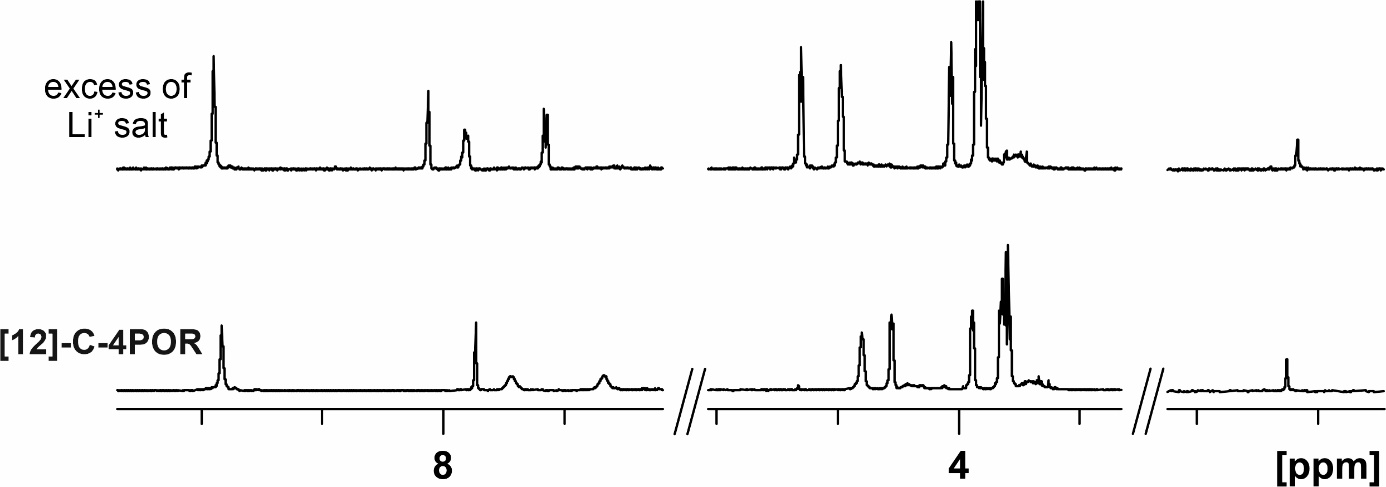


**Figure S20.** The ^1^H NMR spectra (600 MHz, [D_3_]acetonitrile, 300 K) of **[12]-C-4POR** before and after the addition of lithium bis(trifluoromethanesulfonyl)imide (>100 equiv.).


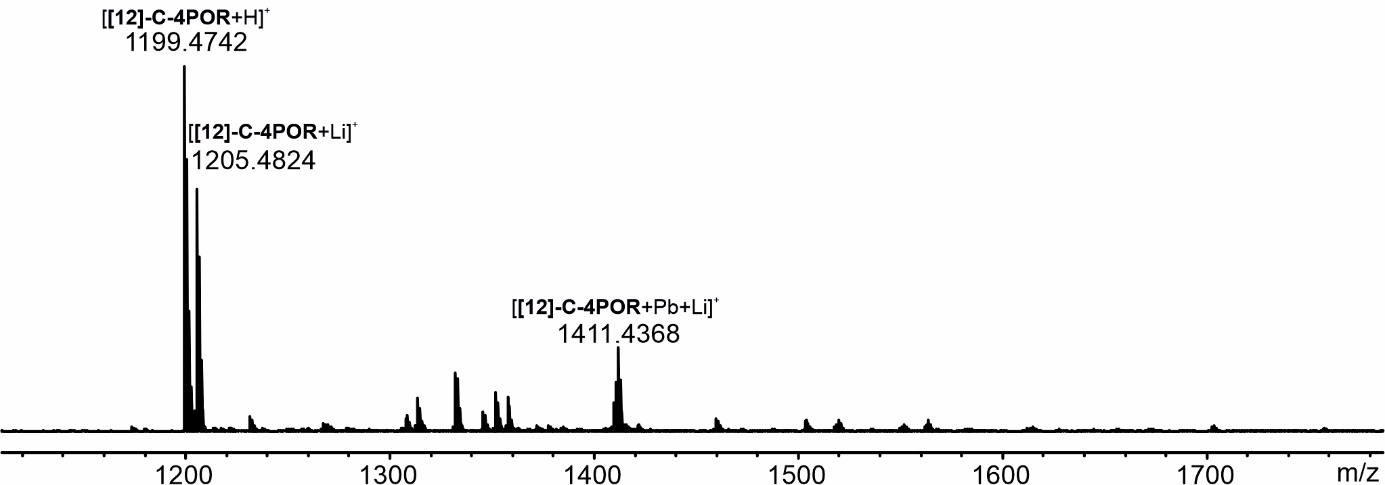


**Figure S21.** The high-resolution mass spectrum of **[12]-C-4POR–Pb** upon the addition of Li^+^ (ESI+, TOF).


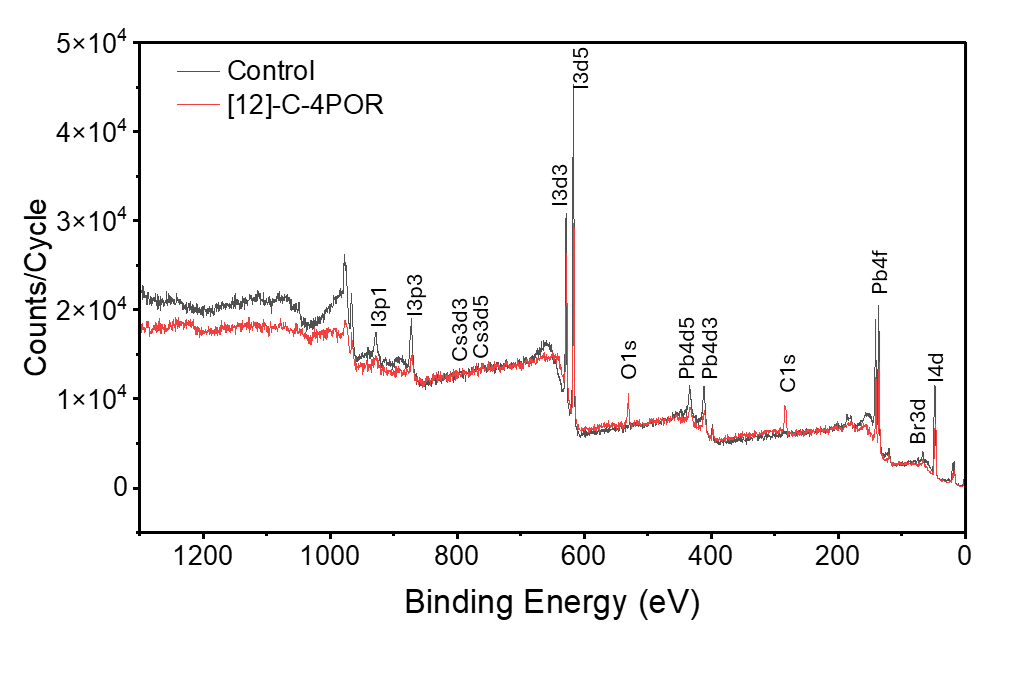


**Figure S22.** The XPS spectra of the control and passivated perovskite films.


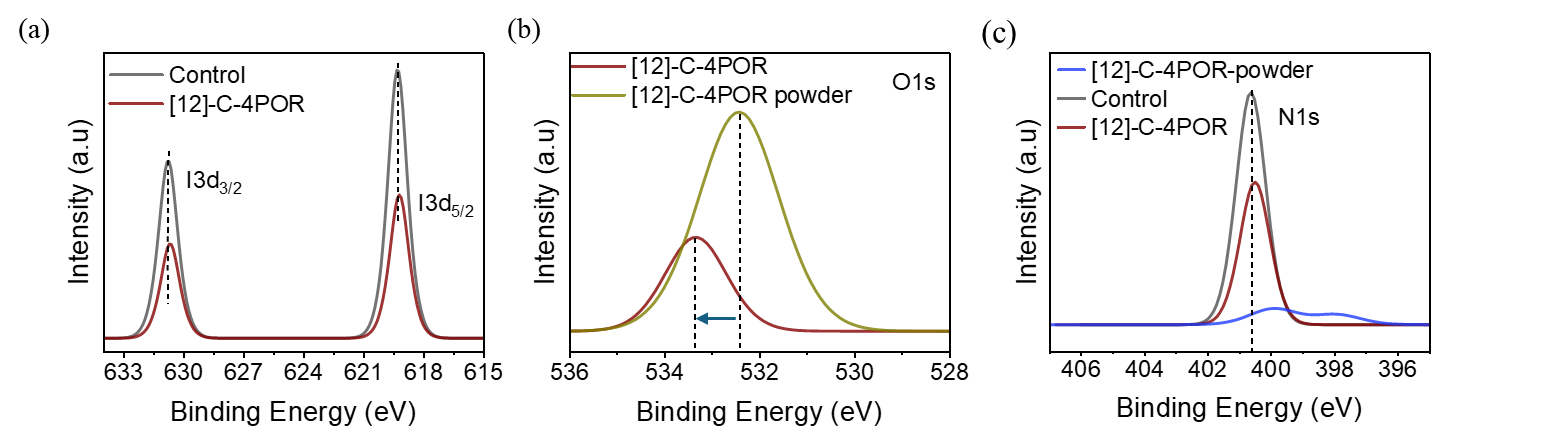


**Figure S23.** XPS spectra of the control and [12]-C-4POR powder and [12]-C-4POR-based perovskite films (a) Iodide (I 3d_5/2_) (b) Oxygen (O1s) (c) N 1s.


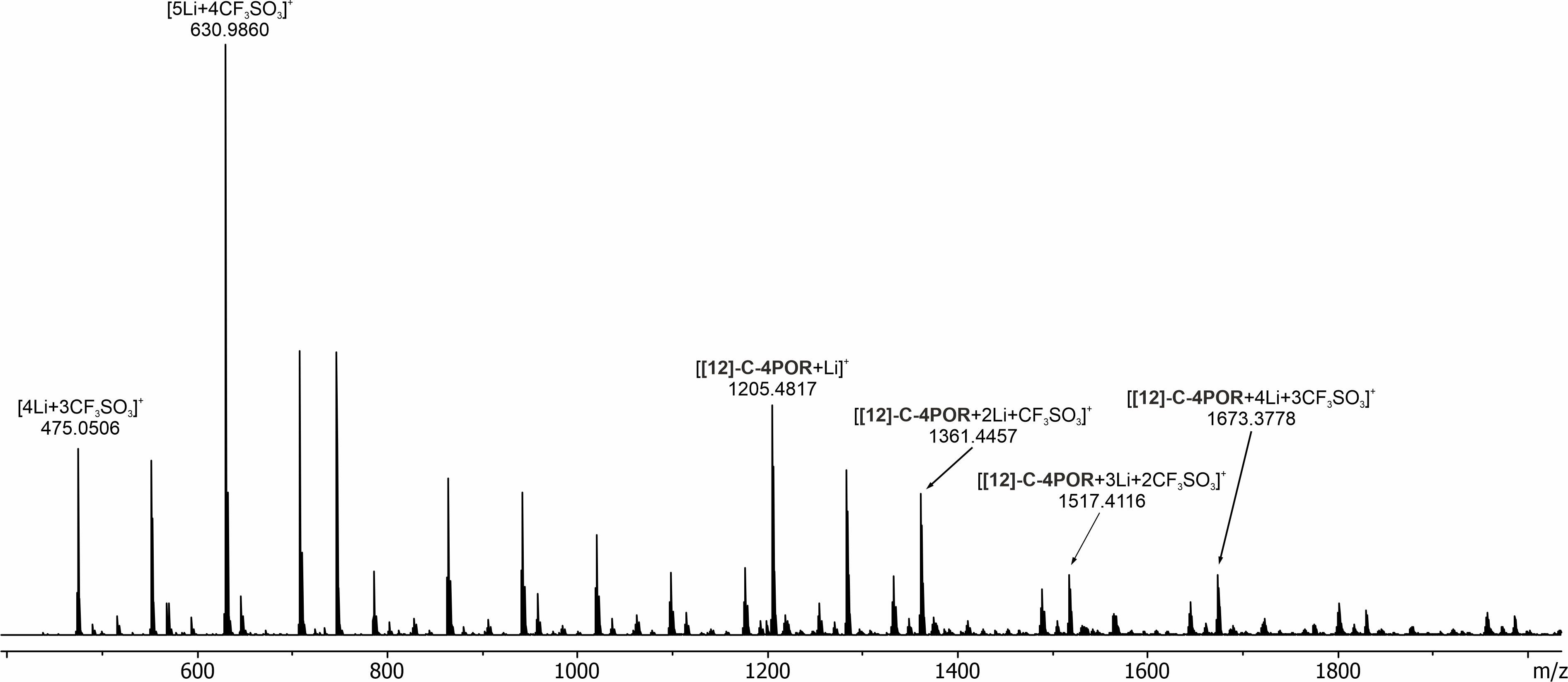


**Figure S24.** The high-resolution mass spectrum of the solution of **[12]-C-4POR** and lithium trifluoromethanesulfonate in DMSO/MeCN/DCM mixture (8:1:1) (ESI+, TOF).


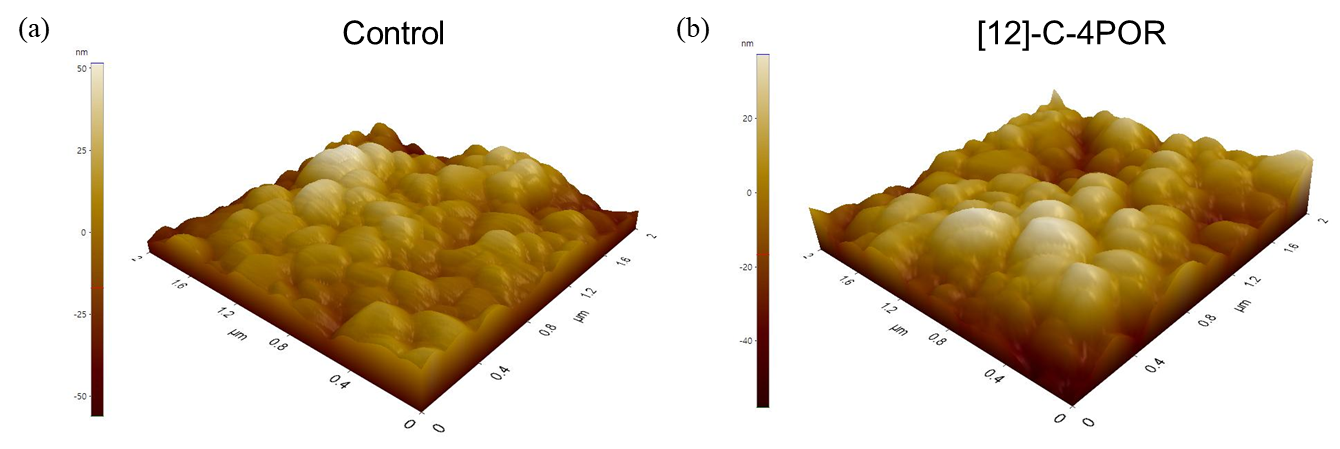


**Figure S25.** 3D AFM images of the control and [12]-C-4POR-based perovskite films.


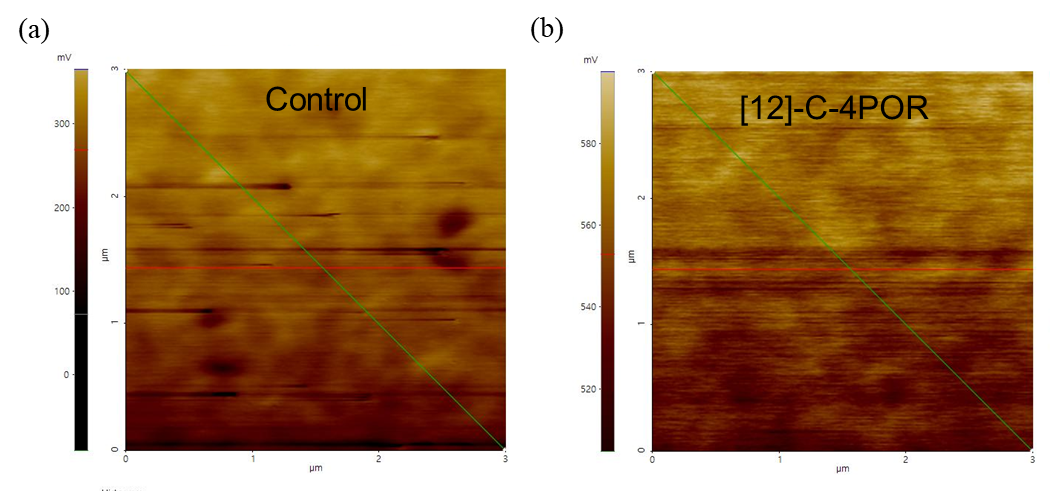


**Figure S26.** KPFM images of the (a) control and (b) [12]-C-4POR-based perovskite films.


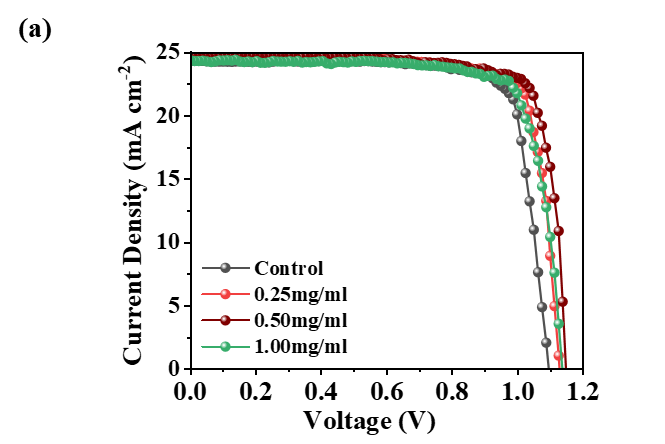


**Figure S27*.*** Current density*−*voltage (*J−V*) curves of the control and [12]-C-4POR-based devices at different concentrations.

**
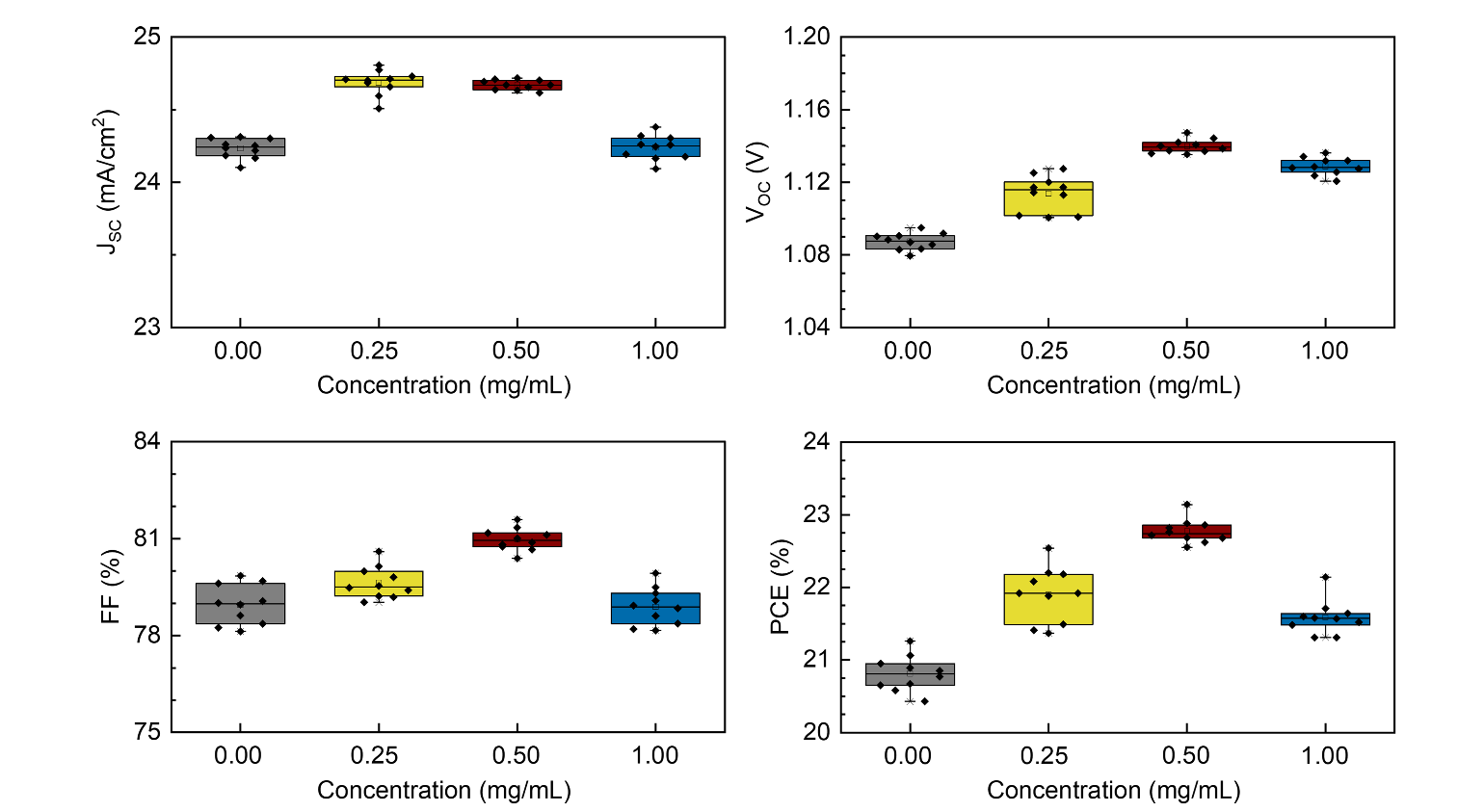
Figure S28*.*** Statistics of the photovoltaic parameter distributions of 10 independent devices under both forward and reverse scan at different [12]-C-4POR concentrations.

***
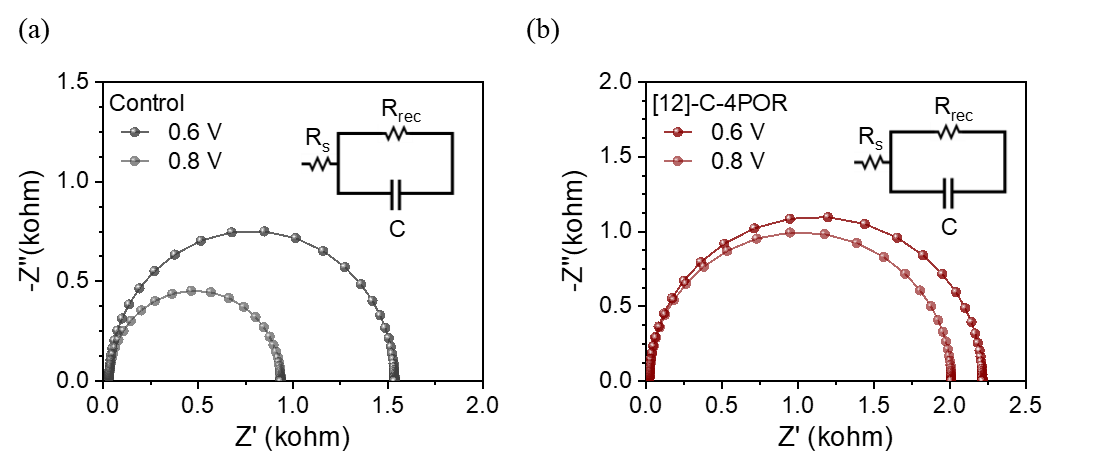
***

**Figure S29*.*** Nyquist plots of impedance spectra for the (a) control and (b) [12]-C-4POR-incorporated devices under a 0.6 and 0.8 V bias.


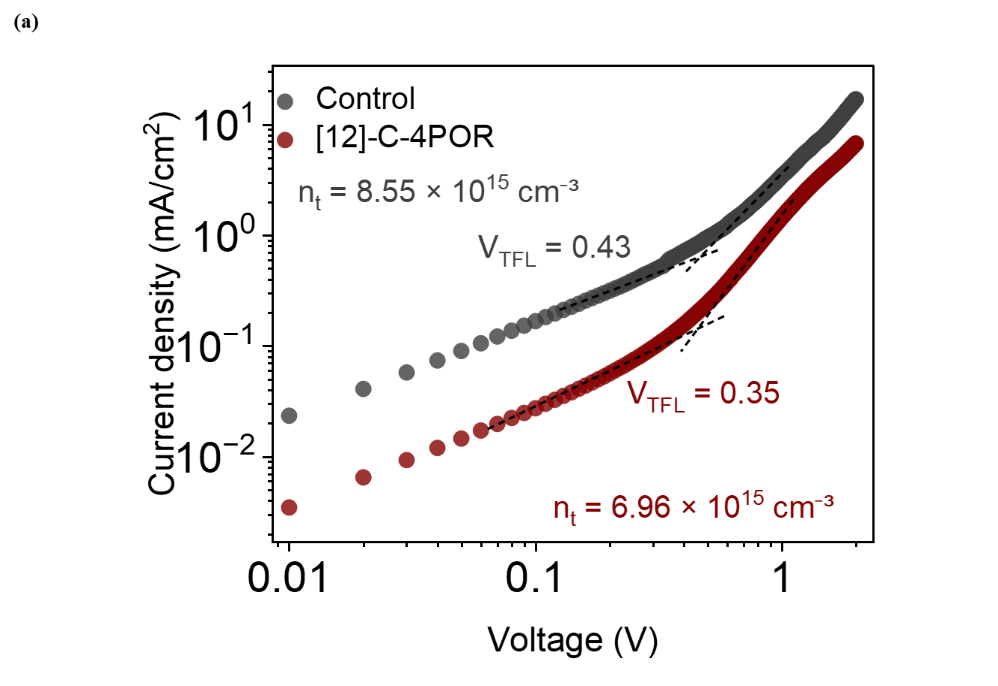


**Figure S30*.*** The current–voltage curves of the hole-only devices employing control and passivated perovskite films.


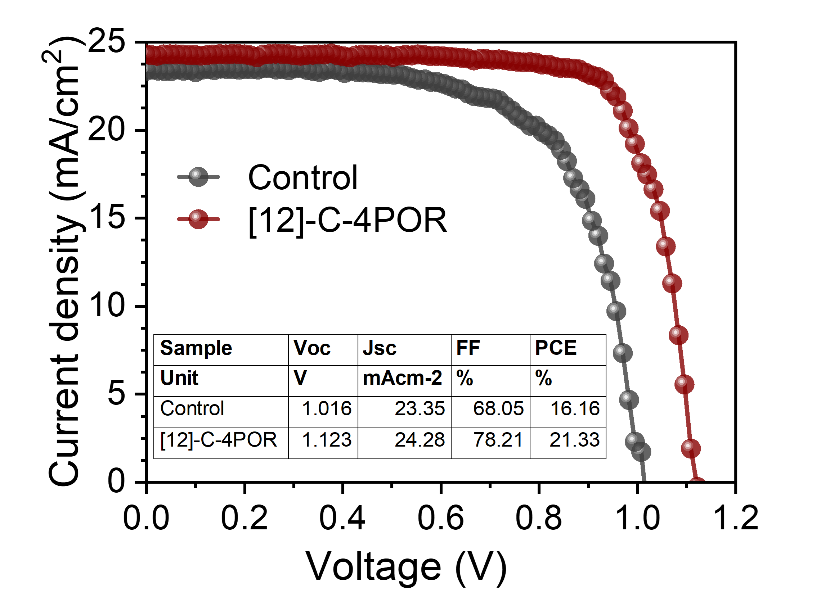


**Figure S31*.*** Current density–voltage (J–V) characteristics of the [12]-C-4POR and control devices measured after 15 days of shelf-life stability.

**Table S1**. Summary of XPS analysis, peak position, full width at half maximum (FWHM), and atomic concentration (%At) of detected elements for the control film.

| Name | Position | FWHM | %At Conc |
| --- | --- | --- | --- |
| Cs 3d 5/2 | 724.93 | 1.17 | **0.16** |
| Cs 3d 3/2 | 738.96 | 1.17 | **0.11** |
|  |  |  | **0.27** |
| I 3d 5/2 | 619.28 | 1.14 | **36.14** |
|  |  |  | **36.14** |
| N 1s | 400.63 | 1.11 | **21.82** |
|  |  |  | **21.82** |
| C 1s A | 284.79 | 1.16 | **5.75** |
| C 1s B | 286.26 | 1.16 | **2.1** |
| C 1s C | 288.38 | 1.16 | **11.74** |
| C 1s C | 289.74 | 1.16 | **1.4** |
|  |  |  | **20.99** |
| Pb 4f 7/2 A | 137.05 | 0.91 | **0.34** |
| Pb 4f 5/2 A | 141.91 | 0.91 | **0.26** |
| Pb 4f 7/2 B | 138.44 | 0.96 | **10.26** |
| Pb 4f 5/2 B | 143.3 | 0.96 | **7.69** |
|  |  |  | **18.55** |
| Br 3d 5/2 | 68.52 | 0.99 | **1.35** |
| Br 3d 3/2 | 69.58 | 0.99 | **0.9** |
|  |  |  | **2.25** |
|  |  |  | **100.02** |

**Table S2**. Summary of XPS analysis, peak position, full width at half maximum (FWHM), and atomic concentration (%At) of detected elements for the [12]-C-4POR-based perovskite films.

| Name | Position | FWHM | %At Conc |
| --- | --- | --- | --- |
| Cs 3d 5/2 | 724.78 | 1.08 | **0.07** |
| Cs 3d 3/2 | 738.79 | 1.08 | **0.04** |
|  |  |  | **0.11** |
| I 3d 5/2 | 619.19 | 1.13 | **11.9** |
|  |  |  | **11.9** |
| O 1s | 533.34 | 1.53 | **13.31** |
|  |  |  | **13.31** |
| N 1s | 400.5 | 1.12 | **8.47** |
|  |  |  | **8.47** |
| C 1s A | 285.04 | 1.34 | **26.2** |
| C 1s B | 286.63 | 1.34 | **26.34** |
| C 1s C | 288.11 | 1.34 | **5.78** |
|  |  |  | **58.32** |
| Pb 4f 7/2 B | 138.38 | 1 | **3.9** |
| Pb 4f 5/2 B | 143.24 | 1 | **2.92** |
|  |  |  | **6.82** |
| Br 3d 5/2 | 68.36 | 1.1 | **0.63** |
| Br 3d 3/2 | 69.44 | 1.1 | **0.42** |
|  |  |  | **1.05** |
|  |  |  | **99.98** |

**Table S3**. Summary of XPS analysis, peak position, full width at half maximum (FWHM), and atomic concentration (%At) of detected elements for the [12]-C-4POR powder.

| Name | Position | FWHM | %At Conc |
| --- | --- | --- | --- |
| O 1s | 532.43 | 2.01 | **18.79** |
|  |  |  | **18.79** |
| N 1s A | 397.9 | 1.77 | **0.42** |
| N 1s B | 399.92 | 1.77 | **0.68** |
|  |  |  | **1.1** |
| C 1s A | 284.8 | 1.91 | **55.62** |
| C 1s B | 286.27 | 1.91 | **13.74** |
|  |  |  | **69.36** |
| Si 2p | 102.3 | 1.96 | **10.75** |
|  |  |  | **10.75** |
|  |  |  | **100** |

**Table S4.** Carrier lifetimes of the control and passivated with **[12]-C-4POR** films.

| Films | τ_1_ (ns) | A_1_ (%) | τ_2_ (ns) | A_2_(%) | τ_av_ (ns) |
| --- | --- | --- | --- | --- | --- |
| Control | 5.74 | 59.76 | 312.63 | 40.23 | 304.48 |
| Passivated | 8.24 | 43.72 | 591.55 | 56.27 | 585.30 |

**Table S5.** Performance of PSCs with different **[12]-C-4POR** concentrations.

| Device |  | V_OC_  (V) | J_SC_  (mA cm^-2^) | FF  (%) | PCE  (%) |
| --- | --- | --- | --- | --- | --- |
| Control | Champion | 1.09 | 24.31 | 79.85 | 21.26 |
|  | Average | 1.08 ± 0.01 | 24.23 ± 0.07 | 78.96 ± 0.62 | 20.81 ± 0.24 |
| 0.25 mg/mL | Champion | 1.13 | 24.81 | 80.60 | 22.54 |
|  | Average | 1.11 ± 0.01 | 24.69 ± 0.09 | 79.64 ± 0.49 | 21.90 ± 0.38 |
| 0.5 mg/mL | **Champion** | **1.15** | **24.72** | **81.59** | **23.14** |
|  | Average | 1.14 ± 0.01 | 24.67 ± 0.03 | 80.97 ± 0.35 | 22.77 ± 0.17 |
| 1 mg/mL | Champion | 1.14 | 24.38 | 79.93 | 22.14 |
|  | Average | 1.13 ± 0.01 | 24.24 ± 0.08 | 78.89 ± 0.58 | 21.59 ± 0.23 |

**Table S6.** Summary of the photovoltaic parameters for the best control and **[12]-C-4POR** based PSCs.

| Device |  | V_OC_  (V) | J_SC_  (mA cm^-2^) | FF  (%) | PCE  (%) | Stabilized PCE (%) | HI (%) |
| --- | --- | --- | --- | --- | --- | --- | --- |
| Control | reverse | 1.09 | 24.31 | 79.85 | 21.26 | 21.18 ± 0.07 | 4.47 |
|  | forward | 1.09 | 24.68 | 75.61 | 20.31 |  |  |
| 0.5 mg/mL | reverse | 1.15 | 24.72 | 81.59 | 23.14 | 23.13 ± 0.08 | 0.86 |
|  | forward | 1.15 | 24.46 | 81.55 | 22.94 |  |  |

**Table S7**. Previously reported crown ether or porphyrin-based molecules for efficient and stable perovskite solar cells.

| Crown ether abbreviations | Passivation | Device Architecture | PCE(%) | References |
| --- | --- | --- | --- | --- |
| DA15C5 | Passivation of the perovskite surface. | ITO/2PACZ/FA_0.9_MA_0.05_Cs_0.05_PbI_3_/DA15C5/C60/BCP/Ag | 22.71 | [2] |
| DB18C6 | Modulation of the perovskite/ETL  interface | FTO/NiOx/MeO-2PACZ/Cs0.05(FA0.17MA0.13)0.95Pb(I0.9Br0.1)3/DB18C6/C60/BCP/Ag | 23.30 | [3] |
| 18C6 | Cation-immobilized phase-stabilized perovskite films. | ITO/SnO2/FACsPbI3/Spiro-OMeTAD/Ag | 19.69 | [4] |
| DB18C6 | Cation-immobilized phase-stabilized perovskite films. | ITO/SnO2/FACsPbI3/Spiro-OMeTAD/Ag | 20.84 | [4] |
| ZnPP | Passivation of the perovskite layer. | (FTO)/SnO2/Perovskite//ZnPP/Spiro-OMeTAD/Ag | 20.80 | [5] |
| A15C5 | Passivation of the perovskite layer. | (FTO)/SnO2/Perovskite/A15C15/Spiro-OMeTAD/Ag | 24.13 | [6] |
| PC12 | Suppression of Li^+^ ion migration | ITO/SnO2/Perovskite+PC15/PC12/Spiro-OMeTAD/Ag | 24.8 | [7] |
| PC15 | Passivation of the uncoordinated Pb^2+^ ions | ITO/SnO2/Perovskite+PC15/PC12/Spiro-OMeTAD/Ag | 24.8 | [7] |
| 12-crown-4 | Suppression of Li^+^ ion migration | ITO/SnO2/Perovskite/12-crown-4+Spiro-OMeTAD/Ag | 23.24 | [8] |
| CS0 | Passivation of the perovskite  layer. | FTO/SnO_2_/PVK/Por/spiro-OMeTAD/Au | 22.14 | [9] |
| CS1 | Passivation of the perovskite layer. | FTO/SnO_2_/PVK/Por/spiro-OMeTAD/Au | 22.37 | [9] |
| CS2 | Passivation of the perovskite layer. | FTO/SnO_2_/PVK/Por/spiro-OMeTAD/Au | 22.17 | [9] |
| ZnPP | Passivation of the perovskite layer. | FTO/SnO_2_/PVK/Por/spiro-OMeTAD/Au | 21.08 | [5] |
| NaCu–Chl | Passivation of the perovskite layer. | FTO/SnO_2_/PVK/Por/spiro-OMeTAD/Au | 20.27 | [10] |
| [12]-C-4POR | **(i)Passivation of the perovskite**  **Layer (ii) Suppression of Li^+^ Migration** | **ITO/SnO_2_/PVK/[12]-C-4POR/spiro-OMeTAD/Au** | **23.14** | **This Work** |

**Table S8.** The EIS parameters of the control and **[12]-C-4POR** based PSCs.

| Devices | R_s_  (Ω) | R_rec_  (kΩ) | C  (F) |
| --- | --- | --- | --- |
| Control | 33.1 | 0.40 | 1.61 x 10^-8^ |
| [12]-C-4POR | 24.3 | 1.94 | 1.71 x 10^-8^ |

**References**

1. Tomar, K. and G. Ramanathan, *New self-assembled archetypes in crown ether substituted Δ Z Phe containing tripeptides.* Journal of Chemical Sciences, 2019. **131**(6): p. 47.

2. Li, B., et al., *Surface Modification with Diaza-Crown Ether for High-Efficiency and Stable Inverted Perovskite Solar Cells.* ACS Applied Materials & Interfaces, 2025. **17**(14): p. 22090-22097.

3. Sui, Y., et al., *Understanding the role of crown ether functionalization in inverted perovskite solar cells.* ACS Energy Letters, 2024. **9**(4): p. 1518-1526.

4. Sun, X., et al., *Dibenzo‐18‐Crown‐6‐Assisted Inhibition of Cation Migration for Stable Perovskite Solar Cells.* Solar RRL, 2022. **6**(9): p. 2200303.

5. Su, K., et al., *In situ graded passivation via porphyrin derivative with enhanced photovoltage and fill factor in perovskite solar cells.* Solar Rrl, 2022. **6**(4): p. 2100964.

6. Chen, X., et al., *Supramolecular aza crown ether modulator for efficient and stable perovskite solar cells.* Advanced Functional Materials, 2024. **34**(17): p. 2311527.

7. Yu, J., et al., *Crown Ethers with Different Cavity Diameters Inhibit Ion Migration and Passivate Defects toward Efficient and Stable Lead Halide Perovskite Solar Cells.* ACS nano, 2024. **18**(33): p. 22533-22547.

8. Shen, Y., et al., *Crowning Lithium Ions in Hole‐Transport Layer toward Stable Perovskite Solar Cells.* Advanced Materials, 2022. **34**(23): p. 2200978.

9. Mai, C.L., et al., *Donor–π–acceptor type porphyrin derivatives assisted defect passivation for efficient hybrid perovskite solar cells.* Advanced Functional Materials, 2021. **31**(7): p. 2007762.

10. Wang, H., et al., *Natural chlorophyll derivative assisted defect passivation and hole extraction for MAPbI3 perovskite solar cells with efficiency exceeding 20%.* ACS Applied Energy Materials, 2022. **5**(2): p. 1390-1396.
